# Uncontrolled Post-Industrial Landfill—Source of Metals, Potential Toxic Compounds, Dust, and Pathogens in Environment—A Case Study

**DOI:** 10.3390/molecules29071496

**Published:** 2024-03-27

**Authors:** Justyna Szulc, Małgorzata Okrasa, Adriana Nowak, Małgorzata Ryngajłło, Joanna Nizioł, Anna Kuźniar, Tomasz Ruman, Beata Gutarowska

**Affiliations:** 1 Department of Environmental Biotechnology, Lodz University of Technology, 90-530 Łódź, Poland; adriana.nowak@p.lodz.pl (A.N.); beata.gutarowska@p.lodz.pl (B.G.); 2 Department of Personal Protective Equipment, Central Institute for Labour Protection—National Research Institute, 90-133 Łódź, Poland; maokr@ciop.lodz.pl; 3 Institute of Molecular and Industrial Biotechnology, Lodz University of Technology, 90-573 Łódź, Poland; malgorzata.ryngajllo@p.lodz.pl; 4 Faculty of Chemistry, Rzeszów University of Technology, 35-959 Rzeszów, Poland; jniziol@prz.edu.pl (J.N.); akuzniar@prz.edu.pl (A.K.); tomruman@prz.edu.pl (T.R.)

**Keywords:** post-industrial landfill, potential toxic substances, heavy metals, metabolome analysis, metagenomics, cytotoxicity, bioaerosols

## Abstract

The aim of this case study was the evaluation of the selected metals’ concentration, potential toxic compound identification, cytotoxicity analysis, estimation of the airborne dust concentration, biodiversity, and number of microorganisms in the environment (leachate, soil, air) of the biggest uncontrolled post-industrial landfills in Poland. Based on the results obtained, preliminary solutions for the future management of post-industrial objects that have become an uncontrolled landfill were indicated. In the air, the PM_1_ fraction dominated, constituting 78.1–98.2% of the particulate matter. Bacterial counts were in the ranges of 9.33 × 10^1^–3.21 × 10^3^ CFU m^−3^ (air), 1.87 × 10^5^–2.30 × 10^6^ CFU mL^−1^ (leachates), and 8.33 × 10^4^–2.69 × 10^6^ CFU g^−1^ (soil). In the air, the predominant bacteria were *Cellulosimicrobium* and *Stenotrophomonas*. The predominant fungi were *Mycosphaerella*, *Cladosporium*, and *Chalastospora*. The main bacteria in the leachates and soils were *Acinetobacter*, *Mortierella*, *Proteiniclasticum*, *Caloramator*, and *Shewanella*. The main fungi in the leachates and soils were *Lindtneria*. Elevated concentrations of Pb, Zn, and Hg were detected. The soil showed the most pronounced cytotoxic potential, with rates of 36.55%, 63.08%, and 100% for the A-549, Caco-2, and A-549 cell lines. Nine compounds were identified which may be responsible for this cytotoxic effect, including 2,4,8-trimethylquinoline, benzo(f)quinoline, and 1-(m-tolyl)isoquinoline. The microbiome included bacteria and fungi potentially metabolizing toxic compounds and pathogenic species.

## 1. Introduction

Recent statistical data indicate that about two billion tons of waste are generated each year. According to the World Bank, this number is projected to reach approximately 3.4 billion tons by 2050 [1]. Poland is one of the main producers of waste in the European Union, generating 123 million tons of municipal and post-production waste in 2020 [2]. Despite the wide availability of modern waste management techniques, storage in landfills remains one of the most frequent forms of waste disposal [3].

Their large size, capacity, and period of operation make landfills one of the most difficult engineering structures to manage [4]. One of the main purposes of landfill operations is to dispose of waste in an environmentally safe manner. This is why modern landfills are designed with appropriate safeguards, in the form of water drainage, degassing systems, and the appropriate sealing of the landfill sides and bottom to minimize leachate migration. According to data from the Central Statistical Office, there are 271 active municipal waste landfills in Poland, with a total area of 1692 ha, as well as 2008 illegal landfills [5].

Despite the growing ecological awareness of the public and legal and financial sanctions against illegal (uncontrolled) waste dumping, the problem of illegal waste dumping is still acute in many countries. Illegal landfills are often created in the areas of forests, lakes, and rivers. The waste in this type of landfill can be extremely diverse and hazardous. It may include construction waste, plastics (packaging, PET bottles, canisters, etc.), clothing and textiles, paper and cardboard (packaging, newspapers, etc.), bulky waste, organic waste (food scraps, etc.), hazardous waste (pharmaceuticals, cans of paints and solvents, bottles of detergents, plant protection products, car fluids, plastic fuel canisters, elements of electrical equipment, etc.), and other waste [1].

Both legal and illegal landfills contribute to environmental pollution. The leachates, gas, and dust generated by landfills pollute the groundwater, soil, and air.

In case of leachate, the greatest threat is posed by illegal landfills, which do not have any barriers such as geomembranes separating them from the ground, waterproofing seals, or an appropriate protection zone. As a result, rainwater penetrates through the landfill, forming leachates containing toxic substances that migrate from the waste. Other sources of leachates include surface water and underground water that flow into the landfill, becoming polluted. The effluents from landfill may contain soluble organic and inorganic substances, as well as heavy metals [6]. Likewise, soil in the vicinity of landfills may contain increased levels of mercury, zinc, copper, arsenic, and lead from hazardous waste, such as paint and varnish residues, batteries, expired pharmaceuticals, and plant protection products [7].

Important pollutants detected in landfill samples, due to their long persistence in the environment and easy migration, are also xenobiotic organic compounds, Persistent Organic Pollutants (POPs) and perfluorinated compounds (PFCs).

Xenobiotic organic compounds, including benzene, toluene, ethylbenzene, xylenes, and halogenated hydrocarbons (e.g., tetrachlorethylene and trichlorethylene), come from both domestic and industrial waste, typically as personal care products, pharmaceuticals, food additives, and packaging materials [8]. POPs include hexachlorocyclohexane (HCH), poly-chlorinated biphenyls (PCBs), hexachlorobenzene (HCB), and hexachlorobutadiene (HCBD) [9]. Likewise, examples of PFCs noted in the landfill environment can be perfluorooctane sulfonate (PFOS) and perfluorooctanoic acid (PFOA) [10]. All these substances are easily bioaccumulated in the food chain, which poses dietary exposure to animals and, ultimately, to humans [8,9,10].

Municipal waste contains large amounts of organic matter and a high moisture content, which is conducive to the development of microorganisms and the generation of odorous compounds such as hydrogen sulfide (H_2_S), ammonia (NH_3_), and volatile organic compounds (VOCs) which are harmful to human health [11,12]. Landfill bioaerosol can consist of over 200 species of microorganisms, predominately *Escherichia*, *Enterobacter*, *Mycobacterium*, *Staphylococcus*, *Streptococcus*, and *Pseudomonas*, as well as many others. The predominant fungi are *Penicillium*, *Alternaria*, *Aspergillus*, and *Rhizopus* [13]. Exposure to landfill bioaerosol can cause many respiratory diseases, allergies, cancers, and infectious diseases, as well as eye and skin irritation [14,15]. Moreover the presence of pharmaceuticals, e.g., antibiotics, results in antibiotic-resistant bacteria and antibiotic-resistant genes which make it increasingly challenging to treat infectious diseases [16].

In turn, asbestos particles can cause asbestosis and cancer when they enter into the human respiratory system [17].

The contamination of leachate and soil in landfills depends on many factors, including the type of waste, method of storage, weather conditions, and the period of operation of the landfill [18].

Post-production waste landfills which initially operated legally may, over time, become unmanaged facilities collecting uncontrolled amounts and types of waste. These illegal landfills are often objects of controversy and fear among the local community. Solving the problems they pose is difficult, due to complicated and unclear legal regulations and the potentially huge costs of extracting and disposing of the waste, especially waste they were not designed to store. 

In the literature, there is a lack of an integrated approach to the problem of multiple pollutants (heavy metals, toxic compounds, dust, and microorganisms) covering large brownfield areas and several environments (air, soil, and leachate) at the same time in the illegal landfills. Identifying this problem using a modern methodology that so far has been used in environmental research studies (the microbiological culture method and laser photometry [19,20]) and the novel approach (AAS—Atomic Absorption Spectrometry; UHPLC-Q-ToF-UHRMS—Ultra-High-Performance Liquid Chromatography-Quadrupole-Time-of-Flight Ultra-High-Resolution Mass Spectrometry; Illumina MiSeq High-Throughput Sequencing and PrestoBlue Assay [11,13,18]) will allow one to collect a large number of results and create a database describing the risks resulting from the landfilling of post-industrial waste. This new database will enable conclusions to be drawn, risk monitoring, and long-term landfill management solutions to be proposed. 

The aim of the study is to assess the comprehensive hazards at one of the largest illegal post-industrial landfills in central Poland, considering the selected metals’ concentration, potential toxic compound identification, cytotoxicity analysis, airborne dust concentration, and the number and biodiversity of microorganisms. Moreover, on the basis of the results obtained, preliminary solutions for the future management of post-industrial objects that have become an uncontrolled landfill were indicated.

## 2. Results and Discussion

### 2.1. Microclimate and Airborne Dust Concentration

**Figure 1:**
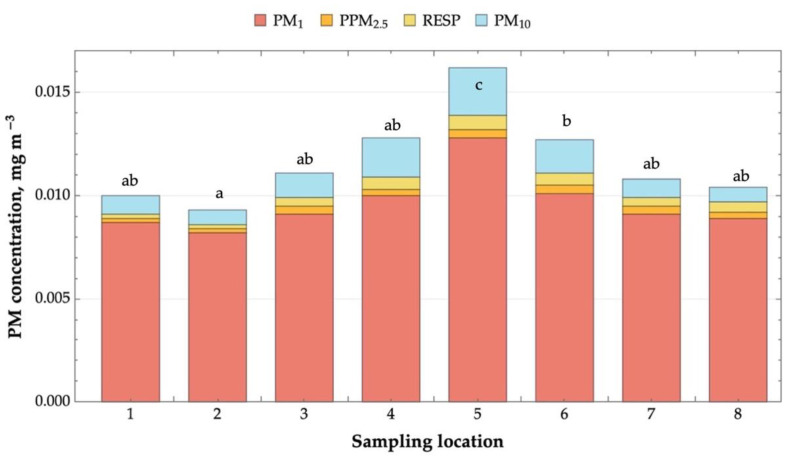
PM concentrations at the tested landfill; 1–8: sampling locations. Different letters (a, b, and c) denote statistically significant differences in PM1 concentrations at different sampling sites (ANOVA followed by Tukey’s test at α = 0.05).

The average temperatures (12.60–15.77 °C), relative humidity (61.07–69.07%), and airflow velocities (0.14–0.32 m s^−1^) in all locations of the tested landfill site were relatively close to the averages for the control location (13.97, 65.70, and 0.26) (Figure S1). The results revealed variations in the PM concentration across the different sampling locations. The smallest particles, PM_1_, constituted a significant share of the total measured dust. While the exact proportions of PM_1_ dust in the total quantity of the measured dust varied between locations (from 78.1% to 88.2%), it remained a significant portion of the total PM (Figure 1). Location 5 consistently showed the highest mean concentrations for all PM size fractions.

It is important to note that exposure to PM, especially fine particles like PM_1_, can pose serious health risks [21]. The European Union has set the environmental threshold for the annual average concentration of dust smaller than 2.5 µm (which includes PM_1_) at 0.025 mg m^−3^, according to its health-based standards and objectives. The PM_1_ concentrations identified in our analysis remained below the established threshold. This indicates that, at least in terms of fine particulate matter, the air quality within the area adheres to the recommended guidelines, suggesting satisfactory air quality.

### 2.2. Metal Concentration in Soil Samples

The concentrations of metals in the soil samples are summarized in Table 1.

In most cases, higher concentrations of metals were detected in the exchangeable (I) fraction than in the carbonate fraction (II). Different concentrations of metals were found depending on the sampling location. The highest concentrations of metals were recorded for locations 5 and 6. After comparing the obtained results with permissible contents in arable land and industrial areas in Poland, according to the Regulation of the Minister of the Environment, 2016, elevated concentrations of Pb were found for soil S6, Zn for soil S5 and S6, and Hg for all tested samples [22]. Soil from an illegal post-industrial landfill can be a source of heavy metal emissions into the environment, especially Pb, Hg, and Zn. The presence of these metals is not surprising, considering the specificity of the studied landfill which was dominated by waste from the textile industry. According to the literature, chemical pollutants from the textile industry include dyes containing metals and carcinogenic amines, pentachlorophenol, chlorine bleaching, halogen carriers, free formaldehyde, fire retardants, and other compounds with toxic potential [23]. According to the literature, up to 70% of the total content of heavy metals can potentially be converted by microorganisms [24,25].

### 2.3. Metabolome Analysis of Soil Samples

**Figure 2:**
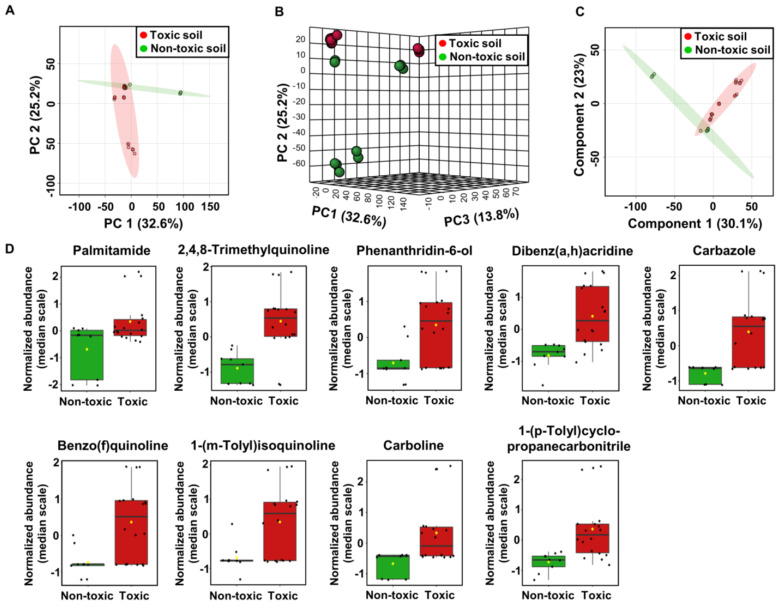
Analysis of soil metabolite profiles created for UHPLC-UHRMS data for distinction of toxic and non-toxic samples, (**A**) 3D PCA (**B**) 3D PLS-DA and (**C**) PLS-DA, which score plot of the toxic (red) and non-toxic (green) soil samples. (**D**) The box–whisker plots of normalized relative abundance data of the most discriminating compound level values observed in toxic and non-toxic samples.

Unsupervised 2D and 3D PCA analysis was used to determine the correlation between the cytotoxicity and chemical composition of the soil samples. The best separation of groups was obtained for principal components 1, 2, and 3 (i.e., PC1, PC2, and PC3), which accounted for 32.6%, 25.2%, and 13.8%, respectively (Figure 2A,B).

The 3D PCA plot showed good differentiation between toxic and non-toxic soil samples taken from the same landfill. Next, a supervised multivariate analysis using PLS-DA was carried out to explore the differences between the toxic and non-toxic soil groups. The PLS-DA score plot indicated good separation between the two groups (Figure 2C). Toxic compounds differentiating both groups were selected based on the VIP plot. Nine compounds, mainly of a synthetic, industrial origin (Appendix A, Table S2, Supplementary Materials), were selected as differential for toxic and non-toxic soil samples. All compounds presented in Table S2 were present in greater amounts in the cytotoxic soils.

Three compounds were found belonging to the class of quinoline derivatives: 2,4,8-trimethylquinoline, benzo(f)quinoline, and 1-(m-tolyl)isoquinoline. The compound that most differentiated both groups was 2,4,8-trimethylquinoline. Methyl derivatives of quinoline are the building blocks of agrochemicals and often have carcinogenic properties [26]. 2,4,8-Trimethylquinoline is included as a carcinogenic in the List of Substances for Trade Unions. Another compound that significantly differentiated the cytotoxic soil samples from the non-cytotoxic samples was palmitamide. This compound is listed as a sensitizer in the Trade Unions’ List of Substances of Concern.

Phenanthridone has been identified as a major mutagenic metabolite of phenanthridine (benzo[c]quinoline) [27]. Due to its similarities in structure to the natural substrate nicotinamide (NAD^+^), phenanthridone can have an immunosuppressive effect as a poly(ADP-ribose) polymerase (PARP) inhibitor [28].

Another compound detected in much greater amounts in toxic soil was dibenz(a,h)acridine. This compound is a five-aromatic-ring polycyclic aromatic hydrocarbon (PAH), which often forms during the incomplete combustion of organic matter. PAHs are common pollutants in the environment that have been detected in all environmental matrices at concentrations of a few ppb to ppm levels. These compounds can be found in gasoline engine exhaust fumes, cigarette smoke, coke oven emissions, charcoal-broiled meats, vegetation near heavily travelled roads, surface water, and soils near hazardous waste sites. It has been shown that the metabolites of this compound are ultimately carcinogenic [29].

Carbazole, which is a tricyclic compound found in coal tar, has the carbon skeleton of fluorene. The carbazole ring system is a structural element of many compounds used in electronics to produce electroluminescent material polymers and dyes. Recently, carbazole derivatives have been shown to exhibit cytotoxic activity. Many carbazoles are known to exert activity through DNA interaction and may cause genetic defects. Like other aromatic amines, carbazole is suspected to cause cancer [30].

Carboline is a heterocyclic compound with an indole ring linked to a pyridine ring. Carboline alkaloids are natural products found in plants, alcoholic beverages, overcooked foods, and tobacco smoke. Carbolines have been reported to exhibit mutagenic and carcinogenic activities in prokaryotic and eukaryotic cells. This is attributed to their ability to intercalate into DNA, resulting in altered DNA replication fidelity and enzymatic activities in DNA-repair processes [31].

### 2.4. Number of Microorganisms in the Air, Leachate, and Soil

**Figure 3:**
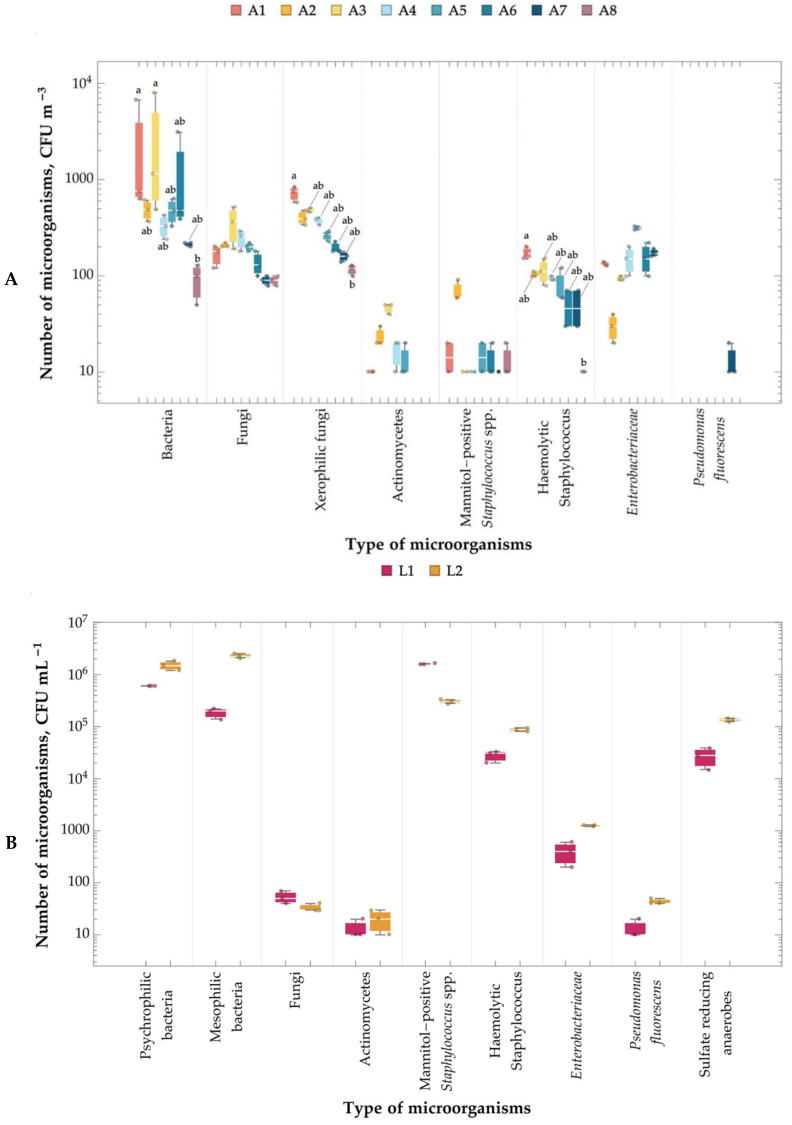

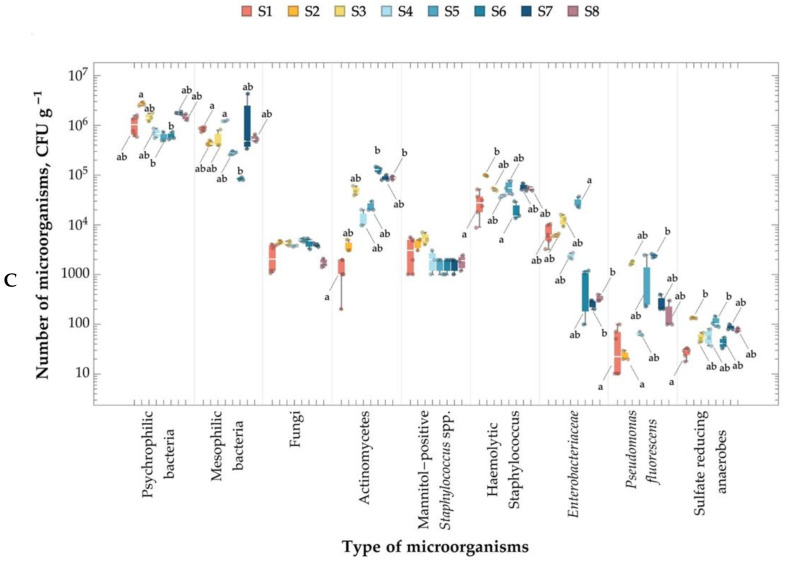
Boxplot showing microbiological contamination at the tested landfill; (**A**) air contamination, A1–A8 air sampling sites; (**B**) leachate in the tested landfill; L1–L2 leachate sampling sites; (**C**) soil contamination at the tested landfill, S1–S8 soil sampling sites. The ends of the boxplot whiskers represent the minimum and maximum values of all the data. Statistically different air and soil samples within the same microorganism group were marked with different lowercase letters (Kruskal–Wallis test followed by Dunn’s post hoc tests at the significance level of 0.05). No statistically significant differences were detected for leachate samples based on the Mann–Whitney U test at α = 0.05.

The number of bacteria in the air at the tested landfill ranged from 9.33 × 10^1^ to 3.21 × 10^3^ CFU m^−3^. The number of fungi was in the range of 9.00 × 10^1^–3.6 × 10^2^ CFU m^−3^. The number of xerophilic fungi was 1.17 × 10^2^–7.23 × 10^2^ CFU m^−3^. Actinomycetes were in the range of 0.00–4.67 × 10^1^ CFU m^−3^. The mean counts of mannitol-positive *Staphylococcus* spp. and hemolytic *Staphylococcus* were 6.67 × 10^0^–7.00 × 10^1^ CFU m^−3^ and 6.67 × 10^0^–1.73 × 10^2^ CFU m^−3^, respectively. *Enterobacteriaceae* bacteria were present in the bioaerosol of the landfill (3.00 × 10^1^–3.13 × 10^2^ CFU m^−3^), but they were not detected in the control air. *Pseudomonas fluorescens* bacteria were detected only in location 7 (1.33 × 10^1^ CFU m^−3^) (Figure 3A, Table S3). Statistical analysis using the Kruskal–Wallis test further confirmed significant differences in the microorganism counts depending on the sampling location for bacteria, xerophilic fungi, and hemolytic staphylococcus (Figure 3A). The data suggest that both the type of microorganism and the sampling location significantly influenced the count of microorganisms in the air samples.

The leachates were dominated by psychrophilic and mesophilic bacteria and mannitol-positive *Staphylococcus* spp., at 1.87 × 10^5^–2.30 × 10^6^ CFU mL^−1^. Lower levels were recorded of hemolytic *Staphylococcus* and sulfate-reducing anaerobes (2.73 × 10^4^–1.35 × 10^5^ CFU mL^−1^). *Enterobacteriaceae* (4.00 × 10^2^–1.27 × 10^3^ CFU mL^−1^) were also present in the leachates, indicating the presence of fecal contamination. Fungi, Actinomycetes, and *Pseudomonas fluorescens* bacteria were the least numerous (1.33 × 10^1^–5.33 × 10^1^ CFU mL^−1^) (Figure 3B, Table S4). The analysis did not reveal statistically significant differences between the sampling locations in terms of microorganism counts.

The numbers of bacteria in the soil ranged from 5.77 × 10^5^ to 2.69 × 10^6^ CFU g^−1^ (psychrophilic bacteria) and from 8.33 × 10^4^ to 1.71 × 10^6^ CFU g^−1^ (mesophilic bacteria). Hemolytic *Staphylococcus* (1.93 × 10^4^–9.73 × 10^4^ CFU g^−1^) and *Enterobacteriaceae* (2.67 × 10^2^–2.74 × 10^4^ CFU g^−1^) were less numerous. The numbers of fungi, actinomycetes, and mannitol-positive *Staphylococcus* spp. Were similar and ranged from 1.20 × 10^3^ to 1.30 × 10^5^ CFU g^−1^. *Pseudomonas fluorescens* and sulfate-reducing anaerobes were the least numerous (2.78 × 10^1^–2.73 × 10^3^ CFU g^−1^) (Figure 3C, Table S4). An analysis of the data revealed a rich diversity of microorganisms in the soil samples, depending on the sampling locations, suggesting a complex and thriving ecosystem. Psychrophilic and mesophilic bacteria tended to have higher counts across all locations. Sulfate-reducing anaerobes, *Pseudomonas fluorescens*, and mannitol-positive *Staphylococcus* spp. had relatively lower counts.

Szulc et al. points out that currently, there are no legal requirements regarding the microbiological quality of air, leachate, or soil in landfill areas [11]. In the literature, it is possible to find data on the number of microorganisms in controlled objects, especially in bioaerosols [32,33,34,35]. Recently, Szulc et al. provided an extensive microbiological characterization of aerosol (69 samples), leachate (33), and soil (63) from four illegal landfills located in central Poland [18]. The authors detected several individual groups of microorganisms, depending on the tested object.

### 2.5. Biodiversity of Air, Leachate, and Soil

**Figure 4:**
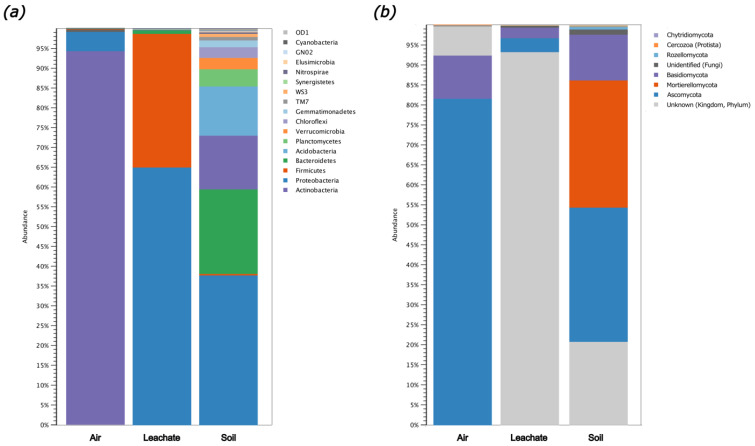
Relative abundance of the identified microorganisms in air, leachate, and soil at the Phylum level. (**a**) Percentage share of bacteria based on the analysis of the V3/V4 region of the gene encoding the 16S rRNA. (**b**) Percentage share of fungi based on the ITS region analysis.

The relative abundance of bacteria identified at the Phylum level, based on the analysis of the V3/V4 region of the 16S rRNA gene, is presented in Figure 4a. The results for fungi and protista, based on an analysis of the ITS region, are presented in Figure 4b. Detailed information on phylogenetic affiliations in the tested samples is given in Table S5 (Supplementary Materials).

Overall, the highest level of diversity of bacteria was identified in the soil sample. Much less diversity was observed in the air and leachate samples. Bacteria belonging to *Actinobacteria* dominated the sample of air, constituting 94% of OUTs (Operational Taxonomic Units). Bacteria classified to *Proteobacteria* constituted the majority of OTUs in the samples of leachate and soil (65% and 38%, respectively). In the leachate sample, a high abundance of Firmicutes was also found (33.7%). In the soil sample, a substantial number of bacteria were found belonging to the following Phyla: *Bacteroidetes* (21.3%), *Actinobacteria* (13.6%), and *Acidobacteria* (12.4%) (Figure 4a). Genera of fungi belonging to *Ascomycota* (81.6%) and *Basidiomycota* (10.8%) predominated in the air sample. In the leachate sample, it was not possible to identify most sequencing readouts (93.3%), although fungi belonging to *Ascomycota* (3.5%) and *Basidiomycota* (2.6%) constituted some share of the identified phyla. In the soil sample, the most abundant phyla were *Ascomycota* (33.6%), *Mortierellomycota* (31.9%), and *Basidiomycota* (11.5%) (Figure 4b).

A detailed analysis showed that the dominant bacterial genera in the air were *Cellulosimicrobium* (94.1%) and *Stenotrophomonas* (3.9%). The dominant bacterial genera in the leachate were *Acinetobacter* (45%), *Proteiniclasticum* (13.5%), *Caloramator* (8.0%), and *Shewanella* (6.7%). In the soil, we found an unknown genus of *Cytophagaceae* (10.0%), *Pseudomonas* (9.3%), an unknown genus of *Saprospiraceae* (4.4%), an unknown genus of *Acidimirobiales* (3.1%), *Myxococcales* (2.7%), an unknown genus of *Xanthomonadaceae* (2.7%), and *Flavobacterium* (2.3%) (Table S5). An analysis of the biodiversity of fungi showed that the following genera dominated in the sample of air: *Mycosphaerella* (56%), *Cladosporium* (5%), and *Chalastospora* (4.9%). In the leachate, the genera identified above 1% included *Taphrina* (1.5%) and *Ustilago* (1.4%). Finally, a much higher level of diversity and abundance of fungi genera were detected in the soil, including *Mortierella* (31.9%), *Lindtneria* (8.0%), *Metarhizium* (4.0%), *Exophiala* (3.8%), an unknown genus of the Pleosporales order (3.5%), and *Penicillium* (3.4%) (Table S5).

The bacteria (*Cellulosimicrobium*, *Stenotrophomonas*, *Acinetobacter*, *Proteiniclasticum*, *Shewanella*, *Pseudomonas*, and *Flavobacterium*) and fungi (*Alternaria*, *Cladosporium*, *Penicillium*, and *Lindtneria*) found in the tested samples had been previously isolated from natural environments, e.g., soil, plant rhizospheress, air, water, sewage, sediments, or marine environments [36,37,38,39,40,41]. Some bacteria were isolated from industrial environments, such as waste and wastewater from the textile industry, especially after the tanning and dyeing processes [37,42]. This is consistent with the present research, as waste from the textile industry (dyes and other chemicals) was stored at the tested landfill.

Some of the identified microorganisms (*Cellulosimicrobium*, *Stenotrophomonas*, and *Mortierella*) can produce a wide spectrum of enzymes (cellulases, laccases, keratinases, and proteases) and secondary metabolites [43,44]. They are capable of decomposing toxic compounds and can be used for the bioremediation of metals. *Cellulosimicrobium* removes iron, zinc, copper, nickel, and cadmium as well as benzo(a)pyrene, chromium (VI), and thorium (IV) from the environment [45]. *Stenotrophomonas* can degrade xenobiotics such as p-nitrophenol and 4-chlorophenol, polycyclic aromatic hydrocarbons, selenium compounds, benzene, and toluene [46,47]. Bacteria from the *Acinetobacter* genus are capable of degrading aromatic hydrocarbons, phenol, diesel, petroleum, and amino acid derivatives [48]. Environmental isolates of *Proteiniclasticum* are responsible for the degradation of herbicides including acetochlor, chloroacetamide, and organoarsenic animal drugs [49,50,51,52]. *Shewanellae* have demonstrated the ability to reduce radionuclides, cobalt, uranium, chromium, mercury, and arsenic, as well as halogenated organic compounds and nitramines [40]. *Pseudomonas* is able to degrade phenolic compounds, pesticides, and petroleum hydrocarbons [53]. These bacteria have been used for the enzymatic dehalogenation of pentachlorophenol from tannery effluent [54]. *Flavobacterium* has the ability to utilize aromatic compounds including naphthalene, biphenyl, and heavy metals [38].

The identified microorganisms are characterized by intrinsic resistance to heavy metals including silver, cadmium, lead, cobalt, zinc, chromium, mercury, and others [46,47]. The dominance of these species in the studied landfill may be related to the active decomposition of post-industrial waste. These microorganisms have high bioremediation potential and *Acinetobacter* has been shown to remove pharmaceutical waste from the environment [55].

The detected microorganisms also have pathogenic potential. Some species of the genera, such as *Cellulosimicrobium*, *Stenotrophomonas*, *Acinetobacter*, *Shewanellae*, and *Pseudomonas*, have been isolated from clinical materials and are associated with human infections including pneumonia, respiratory distress, meningitis, endocarditis, otitis, bacteremia, soft tissue infection, endophthalmitis, septic arthritis, sepsis, wound sepsis, urinary tract infection, and prosthetic joint infections [39,40,47,48,56]. Some of the identified fungi, namely *Mycosphaerella*, *Eryspihe*, *Taphrina*, *Metarhizium*, *Chalastospora*, and *Ustilago*, are known to be plant and animal pathogens [57,58,59].

### 2.6. Cytotoxicity Assessment of Soil Extracts

**Figure 5:**
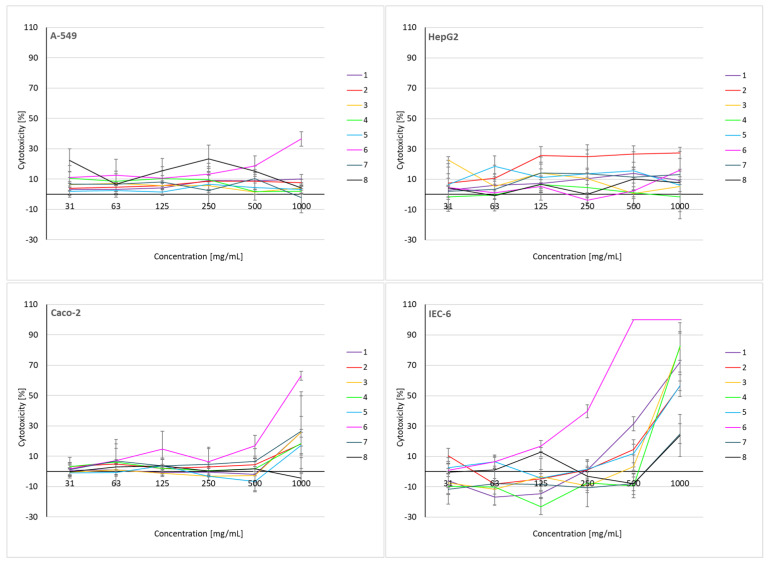
Cytotoxic activity of soil extracts in PrestoBlue assay after 72 h exposition of cells. Each data point represents the mean of the fluorescence values from four replicates. Results are presented as mean ± SD.

As shown in Figure 5, the tested soil extracts demonstrated weak or no cytotoxicity to A-549 lung and HepG2 liver cells. The IC_50_ values could not be estimated for either cell line due to weak cytotoxicity (Table S6, Supplementary Materials). For HepG2 cells, soil extract no. 2 proved to be the most cytotoxic, reaching a cytotoxicity of 27.40% ± 3.70% for the highest concentration tested, i.e., 1000 mg mL^−1^. Soil extract no. 6 demonstrated the strongest cytotoxic potential against three lines, A-549, Caco-2, and A-549, showing cytotoxicity for the highest concentration tested (i.e., 1000 mg mL^−1^) of 36.55% ± 4.80, 63.08% ± 2.90, and 100% ± 0.00%, respectively. The IC_50_ for this extract was 85.81% and 29.24% for Caco-2 and IEC-6 intestinal cells, respectively. Therefore, soil extract no. 6 displayed the highest cytotoxic activity of all the tested extracts against the tested cell lines, especially against IEC-6 normal cells (Table S6). In general, intestinal cells (both lines) were more sensitive to the soil extracts, except for the normal line IEC-6 which was more sensitive than the cancerous Caco-2. For IEC-6, the IC_50_ could be determined for as many as six of the eight tested samples.

The negative results visible in Figure 5 may be due to the excessive metabolic activity or the propagation of cells. This common phenomenon occurs in response to toxic substances as a rescue mechanism against toxic chemicals, especially in the presence of subliminal doses, in order to avoid detrimental stimuli cells which increase metabolism or proliferation [60,61,62]. After prolonged exposure to a given agent, these concentrations may appear to be cytotoxic.

The cytotoxicity of sample no. 6 may be related to the site of its collection. This location showed the lowest average airflow velocity (Figure S1), which can result in the accumulation of pollutants in one place, contributing to soil contamination. The sample taken from location no. 6 showed the strongest cytotoxicity, probably because it was found to contain the highest concentrations of heavy metals. Heavy metals are one of the most common causes of environmental pollution, as well as one of the most dangerous chemical pollutants to health. Lead, cadmium, arsenic, and mercury are especially harmful to human health. Even in trace amounts, these elements are toxic and serve no beneficial function within the body. IARC classifies cadmium as carcinogenic to humans (group 1) [63], lead as probably carcinogenic to humans (group 2A), and mercury as non-carcinogenic (group 3) [64,65]. The heavy metal content is indicative of the typical pollution of an industrial origin. The different cytotoxicities of the other test samples may also be due to the presence of heavy metals in different concentrations and proportions. The effect of soil microorganisms on soil cytotoxicity is rather poorly understood. The results for leachates are not presented because they showed no cytotoxicity in the preliminary tests.

### 2.7. Proposed Solutions and Recommendations including New Insights or Recommendations Based on Our Analysis

Landfills, both organized and unorganized, have a negative impact on the environment; therefore, monitoring and mitigating the effects of illegal waste dumping is becoming the subject of the latest publications. In recent years, dedicated software has been used to control illegal waste storage and identify perpetrators, e.g., based on road surveillance [66]. However, to be effective, this technique requires a high density of roadside monitoring installations and the location of the landfill. The use of mobile electronic devices (mobile phones and tablets), drones, aerial photos as well as satellite and remote-sensing images was also considered to obtain data that, as a result of machine learning, will allow the identification of illegal waste landfill [67].

The analyses carried out in the present work have shown that an uncontrolled post-industrial waste dump poses a serious biological and toxicological threat to the environment and human health. Therefore, a remedial strategy that can be adapted to similar objects should be developed.

The priority in the case of the examined landfill (with an already known location) is to protect it against the influx of new illegally deposited waste. For this purpose, the currently inoperative fence should be repaired and a monitoring system installed in selected places where waste could be delivered. This stage is crucial to start reclamation processes, as well as to minimize the risk of spreading pollutants and, at the same time, protect bystanders from the potential consequences of spontaneous combustions recorded at landfill sites [68]. In this case, patrols and occasional inspections as well as public reports, i.e., in the form of launching a dedicated helpline or a mobile application that allows residents to report undesirable activities in the landfill site are recommended [69]. Enhanced regulatory frameworks, punctuated by stringent enforcement mechanisms, can serve as potent deterrents against illegal waste deposition.

The next step is to decide whether to remove the waste or undertake recultivation in the current state of the landfill. The collected data indicate that a minimum of 40,000 Mg of waste, including hazardous waste, is located in the area of several hectares. From a practical point of view (costs, organizational, technical, and procedural problems), it seems impossible to remove them, especially since the waste is largely covered with earth, and the resulting heaps are systematically covered with vegetation. 

Various substances have already been used successfully for recultivation to improve the physicochemical properties of soils: organic and mineral–organic materials, clay minerals, and organic fertilizers, which can additionally provide nutrients, supporting plant growth, and stimulate the biological activity of the soil. Therefore, in the present case, it seems most reasonable to carry out full reclamation by applying a soil and fertilizer (e.g., sewage sludge), and then planting trees and/or shrubs with a shallow root system. It is recommended that the minimum depth of the covering soil layer should be between 1.25 m for reclamation for agricultural purposes and 2 m for reclamation for tree planting [70].

Moreover, Pusz et al. (2023) emphasizes that the plant cover, especially in the initial reclamation phase, requires careful care. A lack of attention to the health of the plants is a common cause of recultivation failure and may lead to the so-called “secondary wasteland”. High biological activity in the reclamation layer intensifies soil-forming processes and creates favorable conditions for the further development of vegetation, which guarantees the inclusion of the reclaimed facility in the landscape. Floristic and phytosociological studies indicate that over time, the reclaimed site is inhabited by the surrounding vegetation with a large ecological amplitude [71].

It should not be forgotten that there were leachates present in location 1. Therefore, it is necessary to implement a system for the safe collection and treatment of leachate. Different treatments including biological methods such as bioreactors, bioremediation, and hytoremediation, as well as physicochemical techniques (advanced oxidation processes, adsorption, coagulation/flocculation, and membrane filtration) have been involved for this purpose in other studies [72]. The authors recommend the use of integrated methods of the purification and co-treatment of leachates with sewage due to their specificity and low biodegradability [72].

A biodiversity analysis of environmental samples suggests that the microorganisms in the landfill under study include bacteria (*Acinetobacer*, *Caloramator*, *Cellulosimicrobium*, *Flavobacterium*, *Kaistobacter*, *Pseudomonas*, *Proteiniclasticum*, *Shewanella*, and *Stenotrophomonas*) and fungi (*Mortierella*, *Myrmecridium*, and *Exophiala*), which have the appropriate adaptations to survive in the presence of xenobiotics. What is more, they can actively participate in their biological neutralization. 

In our opinion, it is worth trying to isolate these microorganisms in laboratory conditions in order to multiply and construct an appropriate biopreparation that would accelerate the natural decomposition of waste. The use of in situ methods in the case of the current landfill seems to be the most reasonable procedure, and future research directions should aim at intensifying bioremediation with the use of autochthonous strains with laboratory-confirmed effectiveness in neutralizing xenobiotics.

This approach fits into the latest research strategies for searching for environmental microorganisms used in natural bioremediation methods [73,74].

It is worth noting that numerous studies focus on decision-making techniques, such as Geographic Information Systems (GIS) and Multi-Criteria Decision Analysis (MCDA), to support the determination of optimal locations for newly constructed waste landfills [75,76]. However, it is important to highlight that there are currently no established guidelines in the literature regarding decision-making procedures specifically tailored for addressing illegal waste dumps. This gap may be attributed to the individual variability in environmental and human health risks posed by such facilities, as well as the absence of standardized legal regulations.

## 3. Materials and Methods

### 3.1. Landfill Characterization

**Figure 6:**
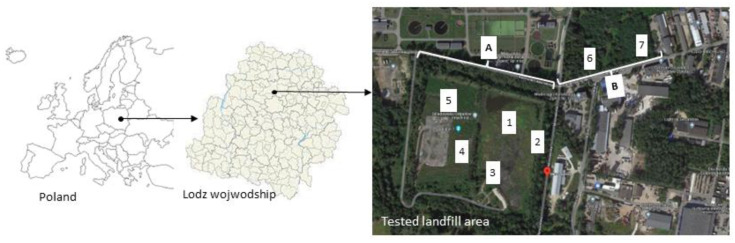
The tested landfill. (**A**) and (**B**)—parts of the landfill (**1**)–(**7**)—sampling locations.

This study concerns an illegal landfill located in central Poland (Łódź Province). The tested landfill and sampling locations are presented in Figure 6 and Table 2.

Part of the landfill (A) operated legally between 1960 and 1986 as the property of a dye production plant and could collect hazardous and non-hazardous waste. The waste was collected later in the years 2007–2013. The landfill covers an area of approx. 2.24 ha. The total amount of deposited waste is not known—according to official data, 40,027.746 Mg were deposited in the years 1995–2006. Later, tens of thousands of Mg of municipal waste were probably dumped illegally. The fence of the landfill is largely destroyed and its area is not secured against unauthorized access. The landfill is bounded on three sides by a causeway (there is no dam on the north side). The slopes are 4–6.5 m high. The bottom of the landfill basin and the slopes are sealed. The sealing consists of native soil developed in the form of sandy loams with a thickness of 5 to 10 m, on which a levelling layer of medium-grained sand is placed. On it, there is a geomembrane (2 mm-thick HDPE foil) and a filtration layer. Drainage pipes are located at the bottom of the post-production waste landfill basin to drain leachate from the landfill area.

The composition of the waste is not fully known. Post-production waste from the dye production plant was stored in the landfill. The waste, to a large extent, is classified as hazardous and was stored in containers, barrels, and various types of containers, covered with a layer of ashes, sand, or rubble, and often with a layer of municipal waste of variable thicknesses.

A significant amount of waste from plants collecting and utilizing hazardous waste, which, for various reasons, was not suitable for processing, was also landfilled. As a rule, they were placed in 200 L barrels, often in original packaging, and poured with concrete.

In addition, Eternit (asbestos) plates with varying degrees of crumbling were deposited at the landfill, which were initially protected with bags or foil against weather conditions. They may be a potential source of respirable asbestos dust entering the atmosphere.

Municipal waste, in turn, was partly transported to an area of over 14 hectares located nearby, where furnace ashes and other industrial waste, including gypsum and layers of ashes, had previously been stored. Their amount was estimated at tens of thousands of tons. This area has been called “geysers” because of the phenomena that occur there, causing micro-fires. The decomposition of municipal waste is an exogenous process in which, among others, methane and hydrogen are produced and, therefore, self-ignition can occur in the landfill. The ongoing decomposition of waste inside the landfill produces a pervasive, unpleasant odor, characterized by compounds like hydrogen sulfide.

Leachate from the landfill was originally discharged to the industrial sewage system and then to the municipal sewage treatment plant. However, after the collapse of the plants, the outflow to the treatment plant was cut off. The lack of insulation of the landfill on its northern side and the closure of the discharge to the sewage treatment plant caused leachates to flow out and accumulate in the land depression at the foot of the northern escarpment of the landfill. They were probably overflowing over the upper boundary of the membrane. The landfill has not been used since 2015 and there is a plant succession in its area.

The second part of the landfill (B), which has never been formally a landfill, is located across the river and covers an area of 4.51 ha. Hazardous and non-hazardous production waste was stored there until the end of the 1990s. Then, they were covered with earth and a forest was planted on the surface. The amount of waste in this part of the landfill is unknown as it is operated illegally [77,78].

**Table 2:** Characteristics of sampling sites in an illegal dumping.

No.		Name	Description	Samples (N)
1	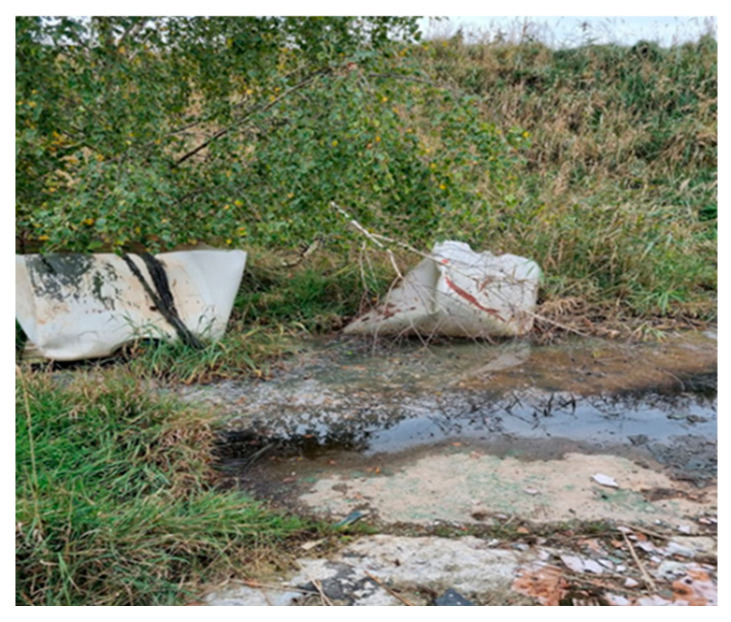	The northern part of the landfill at the foot of the escarpment	At the foot of the slope of the landfill there are abandoned big bags with unidentified content and visible traces of seepage water with greasy stains on the surface. There was an unpleasant “chemical” smell in the air.	A1 (28) S1 (4) L1 (3) L2 (3)
2	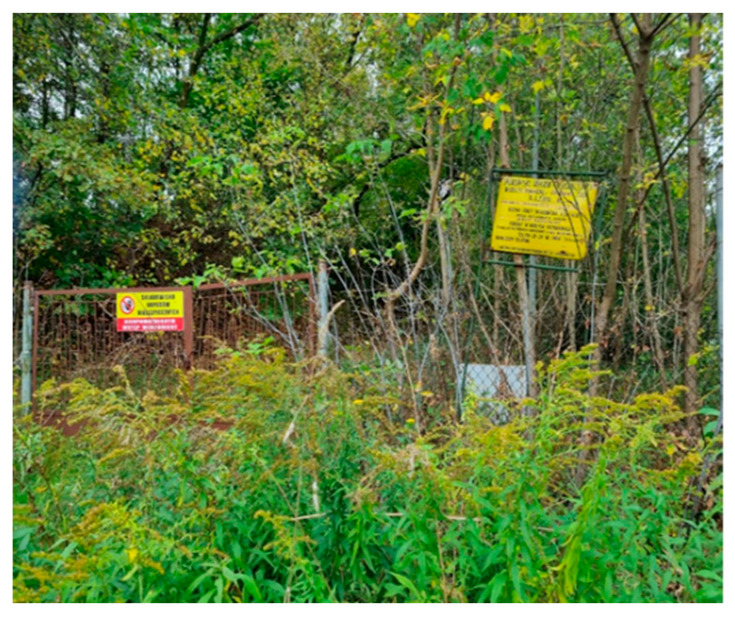	In front of the entrance gate to the landfill site	The area is overgrown with vegetation, a damaged fence is visible	A2 (28) S2 (4)
3	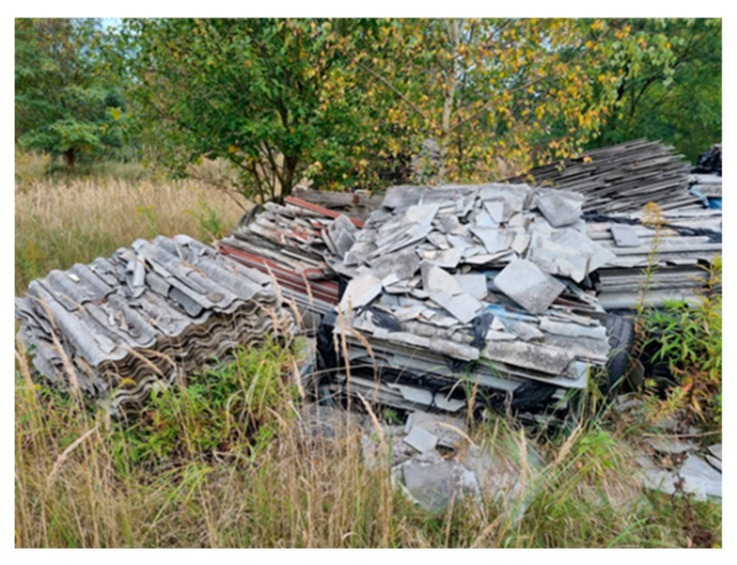	On the left, at the top of the landfill	Visibly broken, unsecured piles of asbestos	A3 (28) S3 (4)
4	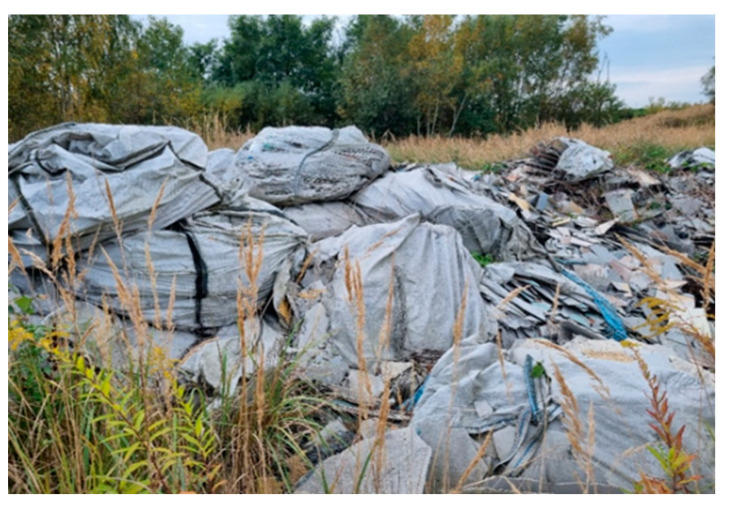	On the right, at the top of the landfill	There is deposited construction waste in bags, visible piles of crushed Eternit	A4 (28) S4 (4)
5	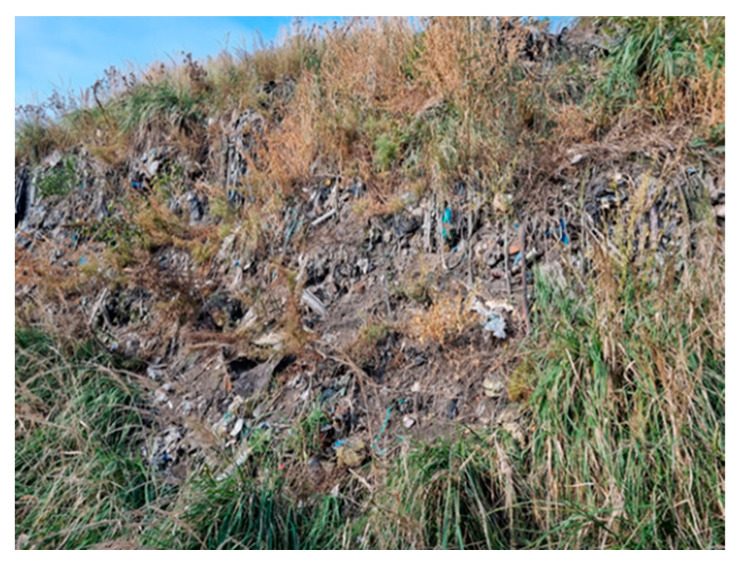	Between heaps of municipal waste	On a heap overgrown with grass, various types of municipal waste are visible	A5 (28) S5 (4)
6	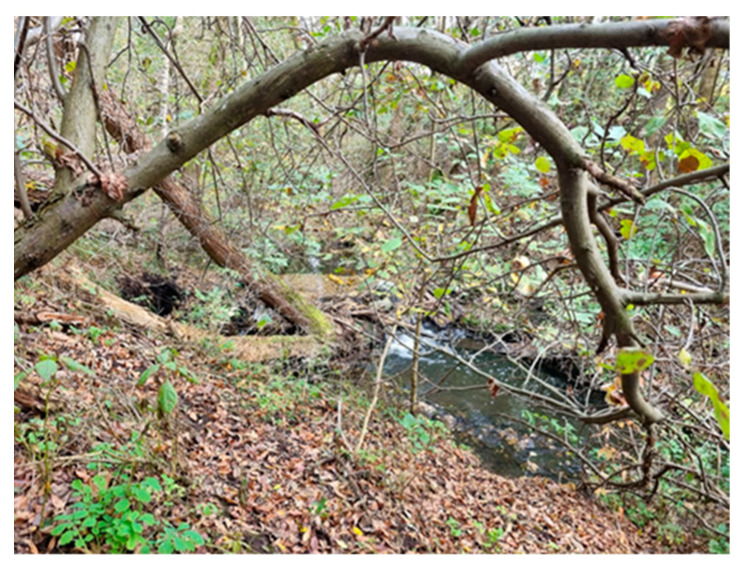	By the river	Plant succession occurs in the study area, greasy stains are visible on the water surface	A6 (28) S6 (4)
7	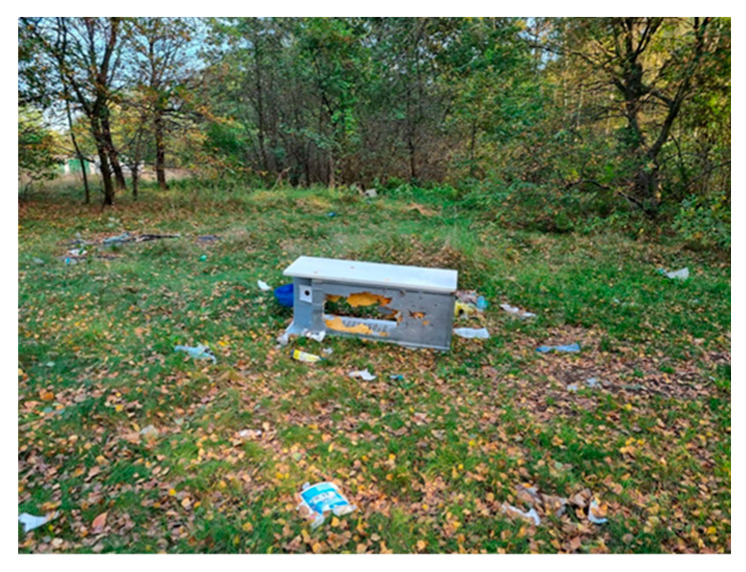	Clearing located near residential buildings	Visible vegetation, scattered municipal waste	A7 (28) S7 (4)
8	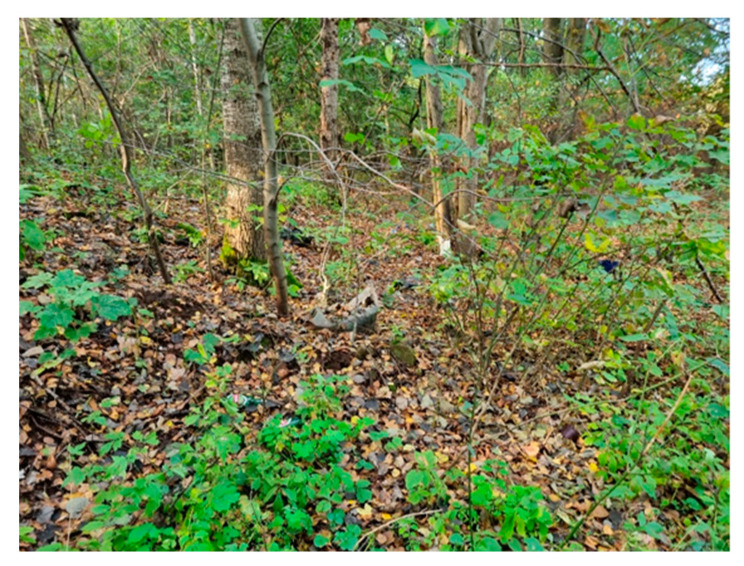	Control samples	5 km from tested landfill site	A8 (27) S8 (4)

A—air; S—soil; L–leachate; N—number of samples.

### 3.2. Microclimate and Airborne Dust Concentration Measurements

We used the VelociCalc^®^ Multi-Function Velocity Meter 954, a thermoanemometer produced by TSI (Shoreview, MN, USA), to determine the temperature, air-flow rate, and relative humidity at the chosen locations. A DustTrak™ DRX Aerosol Monitor 8533, a portable laser photometer also produced by TSI, USA, was employed to evaluate the concentration of the particulate matter (PM). The device can detect particle sizes from 0.1 to 15 μm within a range of 0.001–150 mg m^−3^. Data were collected in triplicate from each site using measuring devices positioned 1.5 m above the ground. The sampling rate was 3 L min^−1^ with intervals of 5 s.

### 3.3. Collection of Environmental Samples

Air, leachate, and soil samples were collected from the landfill area in September 2021. Air samples for the determination of the number of microorganisms were collected using a MAS-100 Eco Air Sampler (Merck Life Science, Warsaw, Poland), according to the EN 13098 standard [79]. Samples of 50, 100, and 200 L of air were collected at a height of 1.5 m from the ground. Atmospheric air samples were also collected 5 km from the landfill (external background). The air (1000 L) for the biodiversity assessment was collected from all sampling sites (1–7) using an AirPort MD 8 (Sartorius, Goettingen, Germany) and sterile gelatine filters (80 mm, 0.3 µL Sartorius, Goettingen, Germany). Leachates and soils were collected in sterile 120 mL plastic containers. The leachates were collected directly in the containers from surface leaks. The soils were sampled using a metal spatula from a depth of 10–20 cm.

### 3.4. Metal Concentration Analysis

Metals, including cadmium (Cd), copper (Cu), (chrome) Cr, (iron) Fe, manganese (Mn), lead (Pb), and zinc (Zn), were analyzed using an atomic absorption spectrometer (PERKIN–ELMER 3100, Shelton, CT, USA) with an air-acetylene flame. The concentrations of Al and Hg were established by the spectrophotometric method (HITACHI U-5100, Tokyo, Japan) using chrome azurol S and dithizone, respectively, as described by [80]. The soil samples were air-dried at an ambient temperature for three weeks, then the samples were ground to a grain diameter of ϕ ≤ 0.25 mm. The weight of a single sample was 10.00 g and during extraction, a universal shaker (time 1 h, speed 120, and amplitude 8) was used. Initially, 50 mL 0.5 mol L^−1^ of MgCl_2_ solution (pH 7) was added to the soil samples. The soils were then leached. Acetate buffer CH_3_COOH/CH_3_COONa, pH 5, was added to the soil residue and the leaching of the soil was continued. Blank extractions were prepared for each sample. Extractions and measurements were carried out in triplicate. Standard AAS (Atomic Absorption Spectroscopy) solutions (1000 mgL^−1^) from Sigma-Aldrich (Poznań, Poland) were used for the analysis.

### 3.5. Metabolome Analysis of Soil Samples

#### 3.5.1. Preparation of Samples

Soil samples (2.0 g) were suspended in 2 mL LC-MS-grade methanol (Sigma-Aldrich, Poznań, Poland), vortexed for 5 min, and left for extraction for 1 h at room temperature. The resulting suspensions were filtered using syringe filters (0.22 µm pores). The soil samples were pipetted into standard HPLC vials and inserted into a Bruker Elute autosampler. The thermostated chamber of the autosampler was set at 5 °C.

#### 3.5.2. UHPLC-Q-ToF-UHRMS Instrumentation

UHPLC-Q-ToF-UHRMS analysis was conducted using a Bruker Elute UHPLC system operated by Hystar 3.3 software, coupled with a Bruker Impact II ESI QToF-MS mass spectrometer (Bruker Daltonik GmbH, Bremen, Germeny; 60,000+ resolution version). The column utilized for the Elute system was a Bruker Intensity Solo, featuring a C18 silica modification with a 1.8 μm particle size and dimensions of 100 × 2.1 mm (length × diameter). The UHPLC column was thermostated at 40 °C. The first mobile (A) phase was water with 0.1% HCOOH. Phase B was acetonitrile with 0.1% HCOOH. The injection volume was 5 μL. Percentage B was 1% (0–2 min), 99% (17–20 min), and 1% (20.1–30 min). The solvent flow rate commenced at 0.25 mL min^−1^ from 0 to 20 min, with a gradual increment to 0.35 mL min^−1^ from 20.1 to 30 min. The internal calibration was based on ions from 10 mM sodium formate pumped into the ESI ion source with a syringe pump at an infusion flow rate of 0.12 mL h^−1^ (solvent: water: isopropanol 1:1 *v*/*v*). All samples were measured in triplicate. The calibration was automatically conducted in Metaboscape using the high-precision calibration (HPC) mode. The instrument was operated in autoMSMS mode. The *m*/*z* range was 50–1500. The CID (Collision-Induced Dissociation) energy value was 30 eV. The CID settings were as follows: absolute area threshold 5000 cts; active exclusion 2; and isolation window for *m*/*z* = 100–4, 300–5, 500–6, and 1000–8. Untargeted annotations were performed in Metaboscape (ver. 2022b) with a criterion of mass deviation (Δ *m*/*z*) under 2 ppm and an mSigma value under 30. MSMS spectra were automatically matched against the following MSMS libraries: the Bruker HMDB 2.0 library, MoNA library [81], and NIST ver. 2020 MS/MS library [82]. The presented methodology was previously published in recent papers [18,83,84,85].

### 3.6. Determination of Number of Microorganisms

The samples of soil (10 g) and leachate (10 mL) were suspended in 90 mL of sterile saline solution (0.85% NaCl). Dilutions were prepared from 10^−1^ to 10^−7^ in triplicate, plated onto the microbiological media, and incubated under the conditions summarized in Table S1. Samples of the air were collected directly onto the microbiological media. Next, the colonies were counted and expressed in CFU m^−3^ (air), CFU g^−1^ (soil), and CFU mL^−1^ (leachate). The result was the arithmetic mean of three independent repetitions.

### 3.7. Determination of Biodiversity

A Genomic Mini AX Bacteria + kit (A&A Biotechnology, Gdańsk, Poland) was employed to extract genomic DNA from the air, soil, and leachate samples. To perform the additional mechanical lysis of the samples, a FastPrep-24 device was used. DNA treatment was carried out using an Anti-Inhibitor Kit (A&A Biotechnology, Gdańsk, Poland) following extraction. A Real-Time PCR was employed to verify the presence of bacterial DNA in the tested samples. The Real-Time PCR was carried out in an Mx3000P thermocycler (Stratagene, La Jolla, CA, USA) using SYBR Green dye as a fluorochrome. The extracted DNA concentration ranged between 2 and 30 µg mL^−1^. Universal primers targeting the 16S rRNA bacterial gene fragment and fungal ITS regions were employed in the reaction [86,87]. The quality and quantity of the DNA eluates were assessed prior to library preparation. The libraries of V3–V4 and ITS amplicons were prepared in accordance with the 16S-Metagenomic-Library-Prep-Guide-15044223-B [88]. A Herculase II Fusion DNA Polymerase Nextera XT Index Kit V2 was used for the two-step PCR. The libraries underwent a quantification and quality assessment following the Illumina qPCR Quantification Protocol Guide. Sequencing was conducted utilizing paired-end technology on an Illumina MiSeq platform (2 × 300 bp) at Macrogen in Seoul, Republic of Korea.

Qualitative and quantitative taxonomic identification was carried out as described in [89], using a CLC Genomic Workbench v. 12 (Qiagen, Redwood City, CA, USA) + Microbial Genomics Module Plugin v. 4.1 (Qiagen). Sequencing data files in FASTQ format were deposited in the NCBI Sequence Read Archive (SRA) under BioProject accession number PRJNA940373 (BioSampleAcc. SAMN33565000-SAMN33565005).

### 3.8. Cytotoxicity Study of the Soil Samples

#### 3.8.1. Cell Propagation and Culturing

The following cell lines were used: Caco-2 (colon adenocarcinoma from humans) cultured in high-glucose DMEM (Dulbecco’s Modified Eagle’s Medium), A-549 cells (lung alveolar adenocarcinoma from humans) in DMEM:Ham’s F12 (1:1, *v*/*v*), Hep-G2 (hepatocellular carcinoma from humans) in Ham’s F12, and IEC-6 (normal small intestine from rats) in low-glucose DMEM:RPMI 1640 (1:1, *v*/*v*). The following supplements were applied: 5 or 10% FBS (foetal bovine serum); 2 or 4 mM GlutaMAX^TM^; and 25 mM HEPES or 0.1 U mL^−1^ insulin. The cells were purchased from the Cell Line Service GmbH and DSMZ German Collection of Microorganisms and Cell Cultures GmbH. The cells were incubated at 37 °C with 5% CO_2_ in a humidified atmosphere for 7–10 days with up to 80% confluence. Next, the cells were washed with 0.1 M PBS (phosphate buffered saline at pH 7.2) and the medium was exchanged. To detach the cells, TrypLE^TM^ Express was used. After performing a cell count using a hemacytometer and determining cell viability with trypan blue (at least 90%), the cells were submitted to further experiments.

#### 3.8.2. Sample Preparation

To 1.0 g of each soil sample, 10 mL of the ready-to-use culture medium (appropriate to the cell line) was added. The sample was mixed and extracted (40 min, ambient air) with shaking (160 rpm.). To eliminate the influence of pH on cytotoxicity, the pH of each extract was adjusted to neutral (pH 7.0 ± 0.2). The extracts were double sterile filtered (with 0.45 and 0.22 µm syringe filters). The initial soil concentration in each extract (stock) was 1000 mg mL^−1^.

#### 3.8.3. PrestoBlue Assay and IC_50_ Calculation

The assay was performed according to manufacturer’s instruction. Cytotoxicity was assessed for water-soluble fractions of the soil samples using PrestoBlue. For this purpose, 5000 (Caco-2, Hep-G2, IEC-6) or 1000 (A-549) cells/well were seeded into 96-well black flat-bottom plates and incubated (24 h, 37 °C, and 5% CO_2_). The following day, the medium was aspirated and then dilutions of the soil extracts were added on the cell monolayers. The final concentrations of the analyzed soil extracts were as follows (mg mL^−1^): 31.3, 62.5, 125, 250, 500, and 1000. The negative control consisted of cells in the vehicle. The exposition time was 72 h. Next, the tested samples were aspirated and PrestoBlue (10% solution in PBS) was added and incubated for a further 2 h. The fluorescence (λ_ex_ 560 nm; λ_em_ 590 nm) was measured in a microplate reader (TriStar2 LB 942, BERTHOLD Technologies GmbH, Bad Wildbad, Germany) and IC_50_ values were estimated from the resulting curves. The mean error of the method is up to 10%.

### 3.9. Statistical Analysis

For the microclimate, airborne dust concentration, and microbial contamination statistical analyses carried out with Statistica 13.1 (Statsoft, Tulsa, OK, USA), descriptive statistics were calculated for all variables of interest. For normally distributed data, ANOVA at α = 0.05 was performed to detect statistically significant differences within the data sets. Tukey’s post hoc procedure was used to compare the means (α = 0.05) in cases where a statistical difference was detected (*p* < 0.05). For data with non-normal distribution, we conducted a Kruskal–Wallis test followed by Dunn’s post hoc tests and a Mann–Whitney U test, all at a significance level of *p* < 0.05.

In cases of metabolite and pathway analysis, all datasets were processed using MetaboAnalyst 5.0 online software [90]. The data were log-transformed, auto-scaled, and sum-normalized. Unsupervised Principal Component Analysis (PCA) and Partial Least Squares Discriminant Analysis were performed on the resulting metabolite profiles (PLS-DA). VIP values of a PLS-DA greater than 1.0 were considered relevant for the classification and retained as significant. The performance of the model was evaluated using cross-validation. The statistical significance of feature-level differences was determined using a paired parametric *t*-test with Mann–Whitney and Bonferroni correction. *p*-values and false discovery rates (FDR; q-value) less than 0.05 were considered statistically significant.

## 4. Conclusions

The adverse environmental effects of uncontrolled landfills are significant. It is vital to conduct extensive research to evaluate the risks posed by these sites, aiming to alter the perception of post-industrial landfills within local communities and determine appropriate strategies for managing illegally accumulated waste. This study reveals that uncontrolled post-industrial waste dumps may pose biological, chemical, and toxicological threats to the environment and human health. The statistically higher number of microorganisms in the air at the landfill, compared to the control air, suggests that the landfill is a source of bioaerosol emissions. These emissions include potentially pathogenic bacteria from the families of *Enterobacteriaceae* and *Pseudomonas fluorescens*, which were also detected in the soil and leachates. A notable concern is the prevalence of potentially pathogenic bacteria and fungi among the identified species, underscoring the biological hazards that an illicit landfill can pose to humans, animals, and plants. The tested soil extracts demonstrated weak cytotoxicity to A-549 lung and HepG2 liver cells and stronger cytoxicity to the IEC-6 cell line. At the same time, our findings indicate that soil can be a source of heavy metal emissions into the environment, especially Pb, Hg, and Zn. The presence of these metals is not surprising, given that the studied landfill contained predominantly waste from the textile industry. Nine compounds were identified which may be responsible for the cytotoxic effect of the soil, most of which were synthetic and of industrial origin, including 2,4,8-trimethylquinoline, benzo(f)quinoline, and 1-(m-tolyl)isoquinoline.

On the basis of the results obtained, in the case of the investigated landfill, a remedial strategy was proposed, based on the literature, including: 1. preventing the influx of new illegally deposited waste through having an appropriate fence and installing a monitoring system; 2. soil remediation by applying an approximately 1.5 m layer of soil and fertilizer (e.g., sewage sludge) on the pile, followed by planting trees and/or shrubs with a shallow root system; 3. the implementation of a system for the safe collection and treatment of leachate, e.g., a system for discharging the leachate to a nearby sewage treatment plant; and 4. the development of a biopreparation using autochthonous strains isolated from the landfill area to neutralize xenobiotics.

## Figures and Tables

**Table 1:** Average concentration of metals detected in soil in the tested landfill (confidence intervals for *p* = 95%).

Sample		Concentration								
		mg kg^−1^						ng kg^−1^		
		Fe	Mn	Pb	Zn	Al	Hg	Cd	Cu	Cr
S1	I	700 ± 25.7	100 ± 5.24	38 ± 0.68	150 ± 6.02	89 ± 0.14	1.1 ± 0.15	4.5 ± 0.18	below LOD	4 ± 0.2
	II	250 ± 0.85	39 ± 1.3	17 ± 1.1	50 ± 2.5	1.5 ± 0.031	3.6 ± 0.40	2.5 ± 0.093	2450 ± 92.02	3.8 ± 0.16
S2	I	435 ± 16.1	88 ± 4.6	16 ± 0.29	16 ± 0.64	8.2 ± 0.24	1.6 ± 0.22	0.8 ± 0.3	4.3 ± 0.15	2.5 ± 0.096
	II	195 ± 0.220	25 ± 1.0	8 ± 0.07	7 ± 0.3	1.3 ± 0.022	6.8 ± 0.45	below LOD	below LOD	3 ± 0.10
S3	I	611 ± 22.1	90 ± 4.7	21 ± 0.38	36 ± 1.3	4.0 ± 0.24	1.2 ± 0.35	2 ± 0.5	7.5 ± 0.29	4 ± 0.2
	II	131 ± 4.90	18 ± 0.85	7 ± 0.15	11 ± 0.32	0.3 ± 0.1	3.9 ± 0.38	below LOD	below LOD	3.3 ± 0.11
S4	I	700 ± 26.4	200 ± 11.4	27 ± 0.48	100 ± 4.14	8.6 ± 0.3	1.6 ± 0.24	1.5 ± 0.065	10.5 ± 0.391	5 ± 0.2
	II	67 ± 0.26	60 ± 1.5	7 ± 0.11	20 ± 0.40	0.3 ± 0.1	7.4 ± 0.56	below LOD	below LOD	3 ± 0.09
S5	I	379 ± 14.3	300 ± 15.6	350 ± 0.632	1425 ± 52.24	3.1 ± 0.24	5.6 ± 0.35	4 ± 0.2	250.0 ± 9.499	12.5 ± 0.488
	II	199 ± 0.221	48 ± 2.0	29 ± 0.12	150 ± 5.25	1.4 ± 0.21	8.9 ± 0.06	1.5 ± 0.078	150 ± 5.478	13.5 ± 0.479
S6	I	800 ± 29.2	400 ± 16.7	285 ± 0.511	1350 ± 50.08	13.7 ± 1.07	2.2 ± 0.16	4.5 ± 0.16	1200 ± 43.92	22.5 ± 1.12
	II	134 ± 5.38	250 ± 0.248	1255 ± 46.04	1100 ± 40.49	0.6 ± 0.2	10.1 ± 0.754	4 ± 0.2	250 ± 9.26	16 ± 0.61
S7	I	970 ± 35.9	150 ± 8.71	11 ± 0.19	20 ± 0.70	8.9 ± 0.21	8.1 ± 0.45	0.5 ± 0.03	7 ± 0.3	2.8 ± 0.16
	II	290 ± 0.353	28 ± 0.11	18 ± 0.15	16 ± 0.66	0.9 ± 0.2	20.6 ± 1.11	below LOD	3.5 ± 0.13	3.5 ± 0.13
S8	I	815 ± 29.7	180 ± 0.914	15 ± 0.27	60 ± 2.3	9.0 ± 0.3	2.7 ±0.80	0.5 ± 0.03	5 ± 0.1	3.5 ± 0.12
	II	112 ± 0.122	23 ± 0.072	6 ± 0.2	8 ± 0.3	0.3 ± 0.08	2.1 ± 0.10	below LOD	below LOD	3 ± 0.12

I—fraction exchangeable, II—fraction associated to carbonates, +/− standard deviation, LOD—Limit of detection.

**Figure A1:**
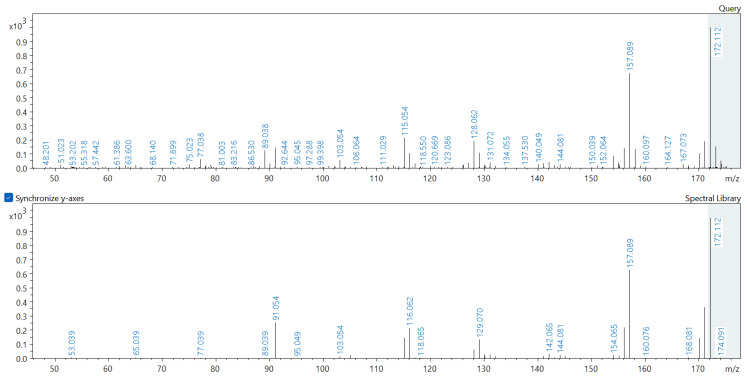
2,4,8-Trimethylquinoline.

**Figure A2:**
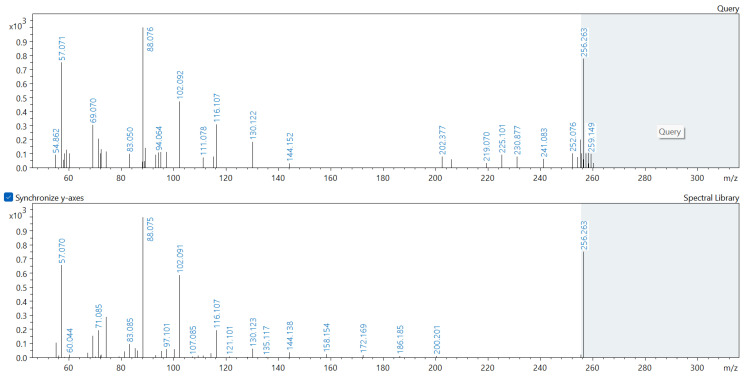
Palmitamide.

**Figure A3:**
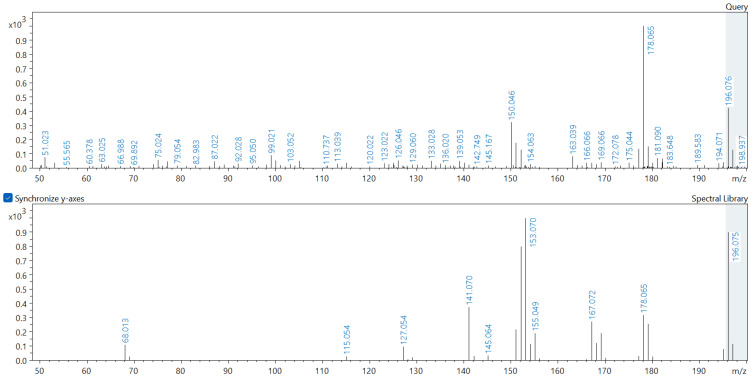
Phenanthridone.

**Figure A4:**
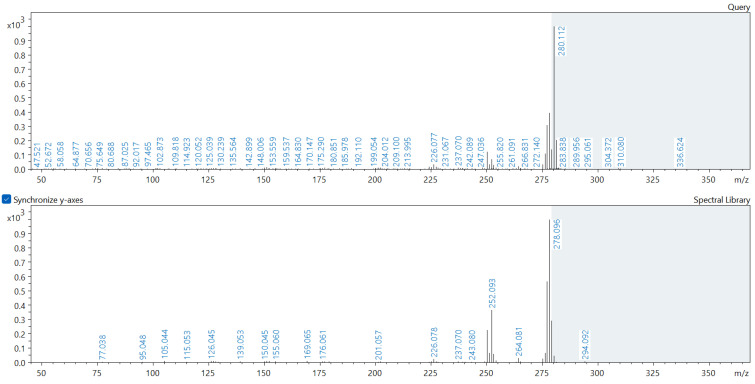
Dibenz(a,H)acridine.

**Figure A5:**
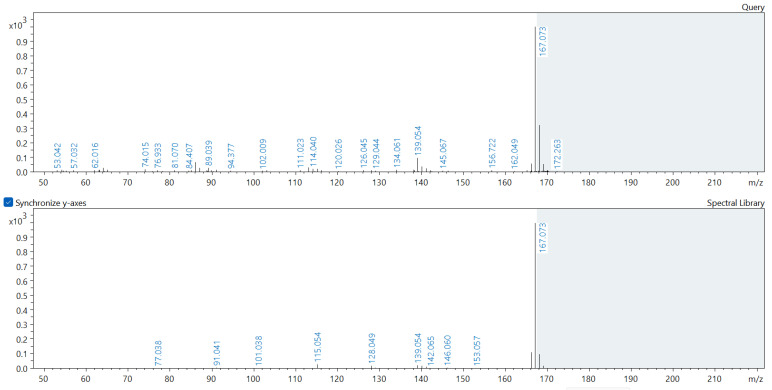
Carbazole.

**Figure A6:**
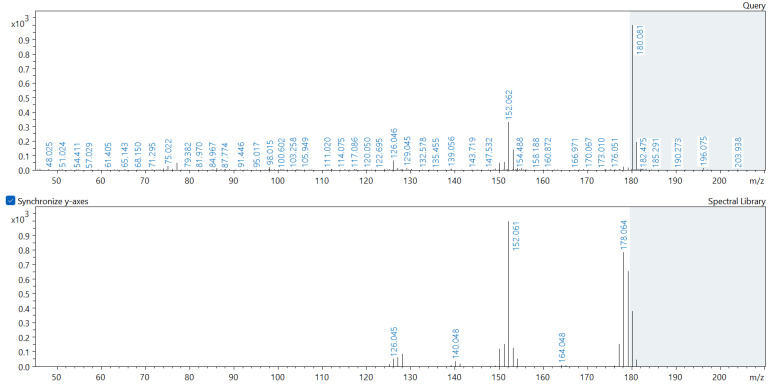
Benzo(f)quinoline.

**Figure A7:**
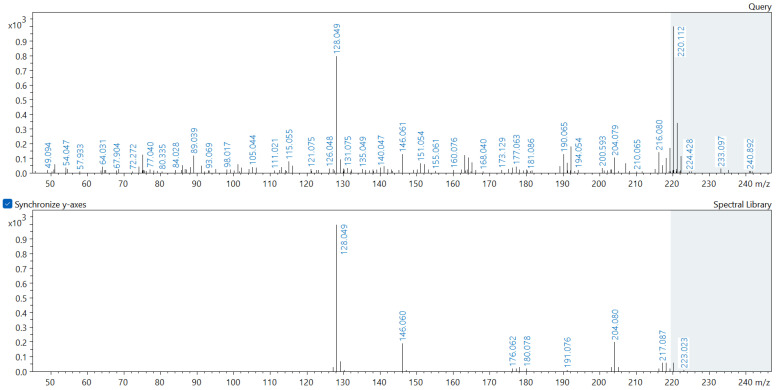
1-(*m*-Tolyl)isoquinoline.

**Figure A8:**
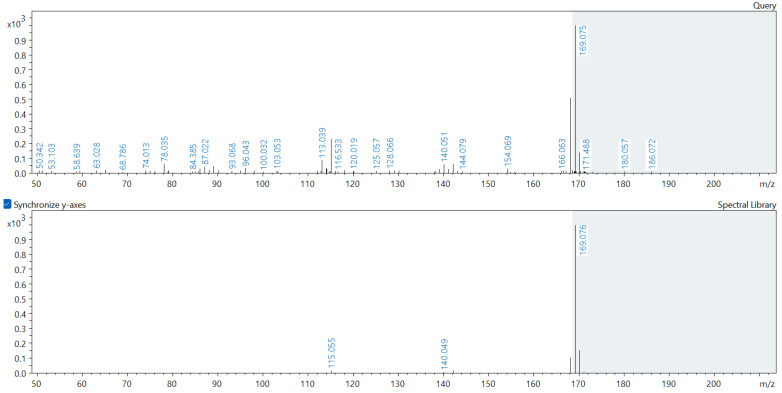
Carboline.

**Figure A9:**
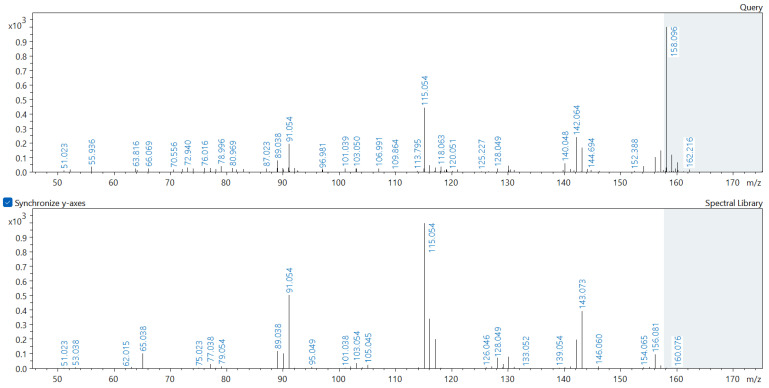
1-(p-Tolyl)cyclopropanecarbonitrile.

## Data Availability

The data presented in this study are available on request from the corresponding author.

## Supplementary Materials

The following supporting information can be downloaded at: https://www.mdpi.com/article/10.3390/molecules29071496/s1, Figure S1: Microclimate parameters in a tested landfill site; (A) temperature, (B) relative humidity, (C) air-flow velocity; a–c: statistically different means were marked with different letters (ANOVA followed by Tukey’s test at α = 0.05), no letters indicate lack of differences; Table S1: Media used for microbiological contamination of air analysis; Table S2: Summary of analysis of toxic compounds found in soil samples (*p*-value < 0.05; FDR < 0.05; VIP > 1; FC > 1.5). All compounds are identified on the basis of high-precursor mass accuracy and also MSMS spectrum and isotopic patterns’ matching; Table S3: Number of microorganisms in the air at a tested, uncontrolled post-industrial landfill; Table S4: Number of microorganisms in the water and soil at a tested, uncontrolled post-industrial landfill; Table S5: Metagenome analysis of microorganisms in landfill environment; Table S6: IC50 [mg/mL] values of soil extracts against cell lines.
