# Supplementary material for: Uncontrolled Post-Industrial Landfill—Source of Metals, Potential Toxic Compounds, Dust, and Pathogens in Environment—A Case Study
Source: Molecules. 2024 Mar 27;29(7):1496. doi: 10.3390/molecules29071496 (PMC11013361; doi:10.3390/molecules29071496)
Supplement: Supplementary file 1 [file molecules-29-01496-s001.zip › Table S1.pdf]

Table S1. Media used for microbiological contamination of air analysis.

Microorganism Type	Medium / Producer	Incubation conditions (temperature /time)
Fungi	Malt Extract Agar (MEA) medium with (0.1%) chloramphenicol / Merck Life Science	25 ± 2 °C / 7 days
Xerophilic fungi	DG18 LAB-AGAR™ (DG18 Agar)* / Biomaxima	25 ± 2 °C / 7 days
Bacteria<br>Psychrofilic bacteria<br>Mesophilic bacteria	Tryptic Soy Agar (TSA) * / Merck Life Science	30 ± 2 °C / 48 h
Haemolytic staphylococcus	Columbia Blood Agar* / Oxoid	37 ± 2 °C / 24–48 h
Actinomycetes	Pochon's agar* / Labomix	25 ± 2 °C / 7 days
mannitol-positive *Staphylococcus* spp.	Chapman Agar* / Merck Life Science	37 ± 2 °C / 24–48 h
Pseudomonas fluorescens	King B medium* / Hi Media Laboratories	30 ± 2 °C / 48 h
Enterobacteriaceae	Violet Red Bile Glucose Agar (VRBG LAB-AGAR) * / Biomaxima	37 ± 2 °C / 24–48 h
sulfate-reducing anaerobes**	Tryptose Sulfite Cycloserine Agar* / Merck Life Science	30 ± 2 °C / 48 h (anaerobic)

* media with (0.2%) nystatin; **determined in soil and leachate samples
