# Supplementary material for: Uncontrolled Post-Industrial Landfill—Source of Metals, Potential Toxic Compounds, Dust, and Pathogens in Environment—A Case Study
Source: Molecules. 2024 Mar 27;29(7):1496. doi: 10.3390/molecules29071496 (PMC11013361; doi:10.3390/molecules29071496)
Supplement: Supplementary file 1 [file molecules-29-01496-s001.zip › Table S2.pdf]

Table S2. Summary of analysis of toxic compounds found in soil samples (P-value < 0.05; FDR < 0.05; VIP > 1; FC > 1.5). All compounds are identified on the basis of high precursor mass accuracy and also MSMS spectrum and isotopic patterns matching.

**Compound**	**Molecular Formula**	**RT [s]**	**Measured *m/z***	**Mass Error [ppm]**	**mSigma**	**MS/MS Score**	**VIP[a]**	**P-value**	**FDR**	**FC[b]**
2,4,8-Trimethyl-quinoline	$C_{12}H_{13}N$	130.2	172.1119	0.89	7.0	903/1000	1.50	3.07E-04	4.54E-03	52.97
Palmitamide	$C_{16}H_{33}NO$	300.9	256.2633	0.37	10.1	917/1000	1.68	8.34E-03	3.40E-02	2.26
Phenanthridone	$C_{13}H_9NO$	182.8	196.0755	1.38	8.5	913/1000	1.34	7.18E-03	3.06E-02	37.31
Dibenz(a,H)acridine	$C_{21}H_{13}N$	295.3	280.1118	0.82	19.7	960/1000	1.22	1.22E-03	7.93E-03	95.59
Carbazole	$C_{12}H_9N$	132.3	168.0807	0.62	8.8	989/1000	1.22	2.06E-03	1.25E-02	117.03
Benzo(f)quinoline	$C_{13}H_9N$	156.7	180.0807	0.46	5.4	930/1000	1.20	4.32E-03	2.07E-02	117.38
1-(m-tolyl)isoquinoline	$C_{16}H_{13}N$	222.1	220.1120	0.89	21.8	847/1000	1.14	7.99E-03	3.29E-02	25.87
Carboline	$C_{11}H_8N_2$	154.0	169.0760	0.20	19.3	948/1000	1.15	1.09E-02	4.14E-02	14.17
1-(p-Tolyl)cyclo-propanecarbonitrile	$C_{11}H_{11}N$	114.3	158.0964	0.84	4.9	877/1000	1.09	5.00E-03	2.32E-02	12.05

[a]VIP scores derived from OPLS-DA model; [b]fold change between toxic and non-toxic samples calculated from the abundance mean values for each group. FC: fold change; FDR: false discovery rate; m/z: mass-to-charge ratio; VIP: variable influence on projection.
