# Supplementary material for: Uncontrolled Post-Industrial Landfill—Source of Metals, Potential Toxic Compounds, Dust, and Pathogens in Environment—A Case Study
Source: Molecules. 2024 Mar 27;29(7):1496. doi: 10.3390/molecules29071496 (PMC11013361; doi:10.3390/molecules29071496)
Supplement: Supplementary file 1 [file molecules-29-01496-s001.zip › Table S3.pdf]

Table S3. Number of microorganisms in the air at an tested uncontrolled postindustrial landfill

Type of microorganisms	Sampling location	Mean	Median	Minimum	Maximum	Standard deviation
Bacteria	1	$2.70x10^3$	$7.60x10^2$	$6.30x10^2$	$6.70x10^3$	$3.47x10^3$
	2	$4.90x10^2$	$4.90x10^2$	$3.70x10^2$	$6.10x10^2$	$1.20x10^2$
	3	$3.21x10^3$	$1.15x10^3$	$4.90x10^2$	$8.00x10^3$	$4.16x10^3$
	4	$3.33x10^2$	$3.30x10^2$	$2.40x10^2$	$4.30x10^2$	$9.50x10^1$
	5	$4.80x10^2$	$4.80x10^2$	$3.30x10^2$	$6.30x10^2$	$1.50x10^2$
	6	$1.32x10^3$	$4.80x10^2$	$3.90x10^2$	$3.10x10^3$	$1.54x10^3$
	7	$2.13x10^2$	$2.20x10^2$	$2.00x10^2$	$2.20x10^2$	$1.15x10^1$
	8	$9.33x10^1$	$1.00x10^2$	$5.00x10^1$	$1.30x10^2$	$4.04x10^1$
Fungi	1	$1.67x10^2$	$1.80x10^2$	$1.20x10^2$	$2.00x10^2$	$4.16x10^1$
	2	$2.07x10^2$	$2.00x10^2$	$2.00x10^2$	$2.20x10^2$	$1.15x10^1$
	3	$3.60x10^2$	$3.70x10^2$	$1.90x10^2$	$5.20x10^2$	$1.65x10^2$
	4	$2.43x10^2$	$2.60x10^2$	$1.80x10^2$	$2.90x10^2$	$5.69x10^1$
	5	$2.00x10^2$	$2.00x10^2$	$1.80x10^2$	$2.20x10^2$	$2.00x10^1$
	6	$1.37x10^2$	$1.30x10^2$	$1.00x10^2$	$1.80x10^2$	$4.04x10^1$
	7	$9.00x10^1$	$9.00x10^1$	$8.00x10^1$	$1.00x10^2$	$1.00x10^1$
	8	$9.00x10^1$	$9.00x10^1$	$8.00x10^1$	$1.00x10^2$	$1.00x10^1$
Xerophilic fungi	1	$7.23x10^2$	$7.60x10^2$	$5.80x10^2$	$8.30x10^2$	$1.29x10^2$
	2	$4.03x10^2$	$3.90x10^2$	$3.40x10^2$	$4.80x10^2$	$7.09x10^1$
	3	$4.83x10^2$	$4.80x10^2$	$4.60x10^2$	$5.10x10^2$	$2.52x10^1$
	4	$3.77x10^2$	$3.90x10^2$	$3.40x10^2$	$4.00x10^2$	$3.21x10^1$
	5	$2.63x10^2$	$2.70x10^2$	$2.30x10^2$	$2.90x10^2$	$3.06x10^1$
	6	$1.97x10^2$	$1.80x10^2$	$1.80x10^2$	$2.30x10^2$	$2.89x10^1$
	7	$1.60x10^2$	$1.60x10^2$	$1.40x10^2$	$1.80x10^2$	$2.00x10^1$
	8	$1.17x10^2$	$1.20x10^2$	$1.00x10^2$	$1.30x10^2$	$1.53x10^1$
Actinomycetes	1	$1.00x10^1$	$1.00x10^1$	$1.00x10^1$	$1.00x10^1$	$0.00x10^0$
	2	$2.33x10^1$	$2.00x10^1$	$2.00x10^1$	$3.00x10^1$	$5.77x10^0$
	3	$4.67x10^1$	$5.00x10^1$	$4.00x10^1$	$5.00x10^1$	$5.77x10^0$
	4	$1.67x10^1$	$2.00x10^1$	$1.00x10^1$	$2.00x10^1$	$5.77x10^0$
	5	$1.33x10^1$	$1.00x10^1$	$1.00x10^1$	$2.00x10^1$	$5.77x10^0$
	6	$3.33x10^0$	$0.00x10^0$	$0.00x10^0$	$1.00x10^1$	$5.77x10^0$
	7	$0.00x10^0$	$0.00x10^0$	$0.00x10^0$	$0.00x10^0$	$0.00x10^0$
	8	$0.00x10^0$	$0.00x10^0$	$0.00x10^0$	$0.00x10^0$	$0.00x10^0$
Mannitol-positive *Staphylococcus* spp.	1	$1.00x10^1$	$1.00x10^1$	$0.00x10^0$	$2.00x10^1$	$1.00x10^1$
	2	$7.00x10^1$	$6.00x10^1$	$6.00x10^1$	$9.00x10^1$	$1.73x10^1$
	3	$6.67x10^0$	$1.00x10^1$	$0.00x10^0$	$1.00x10^1$	$5.77x10^0$
	4	$6.67x10^0$	$1.00x10^1$	$0.00x10^0$	$1.00x10^1$	$5.77x10^0$
	5	$1.00x10^1$	$1.00x10^1$	$0.00x10^0$	$2.00x10^1$	$1.00x10^1$
	6	$1.33x10^1$	$1.00x10^1$	$1.00x10^1$	$2.00x10^1$	$5.77x10^0$
	7	$6.67x10^0$	$1.00x10^1$	$0.00x10^0$	$1.00x10^1$	$5.77x10^0$
	8	$1.33x10^1$	$1.00x10^1$	$1.00x10^1$	$2.00x10^1$	$5.77x10^0$
Haemolytic Staphylococcus	1	$1.73x10^2$	$1.70x10^2$	$1.50x10^2$	$2.00x10^2$	$2.52x10^1$
	2	$1.03x10^2$	$1.00x10^2$	$1.00x10^2$	$1.10x10^2$	$5.77x10^0$

	3	$1.13x10^2$	$1.10x10^2$	$8.00x10^1$	$1.50x10^2$	$3.51x10^1$
	4	$9.33x10^1$	$9.00x10^1$	$9.00x10^1$	$1.00x10^2$	$5.77x10^0$
	5	$8.00x10^1$	$6.00x10^1$	$6.00x10^1$	$1.20x10^2$	$3.46x10^1$
	6	$3.33x10^1$	$3.00x10^1$	$0.00x10^0$	$7.00x10^1$	$3.51x10^1$
	7	$3.33x10^1$	$3.00x10^1$	$0.00x10^0$	$7.00x10^1$	$3.51x10^1$
	8	$6.67x10^0$	$1.00x10^1$	$0.00x10^0$	$1.00x10^1$	$5.77x10^0$
Enterobacteriaceae	1	$1.33x10^2$	$1.30x10^2$	$1.30x10^2$	$1.40x10^2$	$5.77x10^0$
	2	$3.00x10^1$	$3.00x10^1$	$2.00x10^1$	$4.00x10^1$	$1.00x10^1$
	3	$9.33x10^1$	$9.00x10^1$	$9.00x10^1$	$1.00x10^2$	$5.77x10^0$
	4	$1.50x10^2$	$1.50x10^2$	$1.00x10^2$	$2.00x10^2$	$5.00x10^1$
	5	$3.13x10^2$	$3.10x10^2$	$3.00x10^2$	$3.30x10^2$	$1.53x10^1$
	6	$1.57x10^2$	$1.50x10^2$	$1.00x10^2$	$2.20x10^2$	$6.03x10^1$
	7	$1.73x10^2$	$1.70x10^2$	$1.60x10^2$	$1.90x10^2$	$1.53x10^1$
	8	$0.00x10^0$	$0.00x10^0$	$0.00x10^0$	$0.00x10^0$	$0.00x10^0$
Pseudomonas fluorescens	1	$0.00x10^0$	$0.00x10^0$	$0.00x10^0$	$0.00x10^0$	$0.00x10^0$
	2	$0.00x10^0$	$0.00x10^0$	$0.00x10^0$	$0.00x10^0$	$0.00x10^0$
	3	$0.00x10^0$	$0.00x10^0$	$0.00x10^0$	$0.00x10^0$	$0.00x10^0$
	4	$0.00x10^0$	$0.00x10^0$	$0.00x10^0$	$0.00x10^0$	$0.00x10^0$
	5	$0.00x10^0$	$0.00x10^0$	$0.00x10^0$	$0.00x10^0$	$0.00x10^0$
	6	$0.00x10^0$	$0.00x10^0$	$0.00x10^0$	$0.00x10^0$	$0.00x10^0$
	7	$1.33x10^1$	$1.00x10^1$	$1.00x10^1$	$2.00x10^1$	$5.77x10^0$
	8	$0.00x10^0$	$0.00x10^0$	$0.00x10^0$	$0.00x10^0$	$0.00x10^0$
