# Supplementary material for: Uncontrolled Post-Industrial Landfill—Source of Metals, Potential Toxic Compounds, Dust, and Pathogens in Environment—A Case Study
Source: Molecules. 2024 Mar 27;29(7):1496. doi: 10.3390/molecules29071496 (PMC11013361; doi:10.3390/molecules29071496)
Supplement: Supplementary file 1 [file molecules-29-01496-s001.zip › Table S4.pdf]

Table S4. Number of microorganisms in the water and soil at an tested uncontrolled postindustrial landfill

Medium	Type of microorganisms	Sampling location	Mean	Median	Minimum	Maximum	Standard deviation
Water	Psychrophilic bacteria	1	$6.03x10^5$	$6.00x10^5$	$6.00x10^5$	$6.10x10^5$	$5.77x10^3$
		2	$1.50x10^6$	$1.48x10^6$	$1.20x10^6$	$1.81x10^6$	$3.05x10^5$
	Mesophilic bacteria	1	$1.87x10^5$	$2.00x10^5$	$1.40x10^5$	$2.20x10^5$	$4.16x10^4$
		2	$2.30x10^6$	$2.25x10^6$	$2.10x10^6$	$2.54x10^6$	$2.24x10^5$
	Fungi	1	$5.33x10^1$	$5.00x10^1$	$4.00x10^1$	$7.00x10^1$	$1.53x10^1$
		2	$3.33x10^1$	$3.00x10^1$	$3.00x10^1$	$4.00x10^1$	$5.77x10^0$
	Actinomycetes	1	$1.33x10^1$	$1.00x10^1$	$1.00x10^1$	$2.00x10^1$	$5.77x10^0$
		2	$2.00x10^1$	$2.00x10^1$	$1.00x10^1$	$3.00x10^1$	$1.00x10^1$
	Mannitol-positive *Staphylococcus* spp.	1	$1.61x10^6$	$1.60x10^6$	$1.59x10^6$	$1.63x10^6$	$2.08x10^4$
		2	$3.03x10^5$	$3.00x10^5$	$2.80x10^5$	$3.30x10^5$	$2.52x10^4$
	Haemolytic Staphylococcus	1	$2.80x10^4$	$3.10x10^4$	$2.00x10^4$	$3.30x10^4$	$7.00x10^3$
		2	$8.80x10^4$	$8.80x10^4$	$8.10x10^4$	$9.50x10^4$	$7.00x10^3$
	Enterobacteriaceae	1	$4.00x10^2$	$4.00x10^2$	$2.00x10^2$	$6.00x10^2$	$2.00x10^2$
		2	$1.27x10^3$	$1.30x10^3$	$1.20x10^3$	$1.30x10^3$	$5.77x10^1$
	Pseudomonas fluorescens	1	$1.33x10^1$	$1.00x10^1$	$1.00x10^1$	$2.00x10^1$	$5.77x10^0$
		2	$4.33x10^1$	$4.00x10^1$	$4.00x10^1$	$5.00x10^1$	$5.77x10^0$
	Sulfate reducing anaerobes	1	$2.73x10^4$	$2.80x10^4$	$1.50x10^4$	$3.90x10^4$	$1.20x10^4$
		2	$1.35x10^5$	$1.36x10^5$	$1.25x10^5$	$1.45x10^5$	$1.00x10^4$
Soil	Psychrophilic bacteria	1	$1.06x10^6$	$1.07x10^6$	$6.00x10^5$	$1.58x10^6$	$4.31x10^5$
		2	$2.69x10^6$	$2.63x10^6$	$2.50x10^6$	$2.95x10^6$	$2.32x10^5$
		3	$1.45x10^6$	$1.41x10^6$	$1.20x10^6$	$1.74x10^6$	$2.72x10^5$
		4	$7.07x10^5$	$6.80x10^5$	$5.70x10^5$	$8.70x10^5$	$1.52x10^5$
		5	$5.77x10^5$	$5.10x10^5$	$4.90x10^5$	$7.30x10^5$	$1.33x10^5$
		6	$6.07x10^5$	$5.50x10^5$	$5.40x10^5$	$7.30x10^5$	$1.07x10^5$
		7	$1.75x10^6$	$1.76x10^6$	$1.65x10^6$	$1.85x10^6$	$1.00x10^5$
		8	$1.48x10^6$	$1.55x10^6$	$1.26x10^6$	$1.64x10^6$	$2.00x10^5$
	Mesophilic bacteria	1	$8.40x10^5$	$8.55x10^5$	$7.50x10^5$	$9.00x10^5$	$6.23x10^4$
		2	$4.33x10^5$	$4.20x10^5$	$3.90x10^5$	$4.90x10^5$	$5.13x10^4$
		3	$5.30x10^5$	$4.00x10^5$	$3.90x10^5$	$8.00x10^5$	$2.34x10^5$
		4	$1.21x10^6$	$1.20x10^6$	$1.18x10^6$	$1.26x10^6$	$4.16x10^4$
		5	$2.80x10^5$	$2.80x10^5$	$2.50x10^5$	$3.10x10^5$	$3.00x10^4$
		6	$8.33x10^4$	$8.00x10^4$	$8.00x10^4$	$9.00x10^4$	$5.77x10^3$
		7	$1.71x10^6$	$4.80x10^5$	$3.40x10^5$	$4.30x10^6$	$2.25x10^6$
		8	$5.44x10^5$	$5.20x10^5$	$4.72x10^5$	$6.40x10^5$	$8.65x10^4$
	Fungi	1	$2.50x10^3$	$2.35x10^3$	$1.10x10^3$	$4.10x10^3$	$1.47x10^3$
		2	$4.53x10^3$	$4.60x10^3$	$4.10x10^3$	$4.90x10^3$	$4.04x10^2$
		3	$4.33x10^3$	$4.40x10^3$	$3.90x10^3$	$4.70x10^3$	$4.04x10^2$
		4	$3.77x10^3$	$3.80x10^3$	$3.60x10^3$	$3.90x10^3$	$1.53x10^2$
		5	$4.97x10^3$	$5.00x10^3$	$4.50x10^3$	$5.40x10^3$	$4.51x10^2$
		6	$4.43x10^3$	$4.80x10^3$	$3.30x10^3$	$5.20x10^3$	$1.00x10^3$
		7	$4.00x10^3$	$4.10x10^3$	$3.60x10^3$	$4.30x10^3$	$3.61x10^2$
		8	$1.73x10^3$	$1.70x10^3$	$1.40x10^3$	$2.10x10^3$	$3.51x10^2$

Actinomycetes	1	$1.20x10^3$	$1.00x10^3$	$2.00x10^2$	$2.00x10^3$	$6.93x10^2$
	2	$3.67x10^3$	$3.00x10^3$	$3.00x10^3$	$5.00x10^3$	$1.15x10^3$
	3	$4.83x10^4$	$4.50x10^4$	$4.00x10^4$	$6.00x10^4$	$1.04x10^4$
	4	$1.33x10^4$	$1.00x10^4$	$1.00x10^4$	$2.00x10^4$	$5.77x10^3$
	5	$2.33x10^4$	$2.00x10^4$	$2.00x10^4$	$3.00x10^4$	$5.77x10^3$
	6	$1.30x10^5$	$1.30x10^5$	$1.10x10^5$	$1.50x10^5$	$2.00x10^4$
	7	$8.67x10^4$	$8.00x10^4$	$8.00x10^4$	$1.00x10^5$	$1.15x10^4$
	8	$8.83x10^4$	$8.80x10^4$	$8.10x10^4$	$9.60x10^4$	$7.51x10^3$
Mannitol-positive *Staphylococcus* spp.	1	$3.20x10^3$	$3.35x10^3$	$1.00x10^3$	$5.50x10^3$	$2.09x10^3$
	2	$4.33x10^3$	$5.00x10^3$	$3.00x10^3$	$5.00x10^3$	$1.15x10^3$
	3	$5.33x10^3$	$5.00x10^3$	$4.00x10^3$	$7.00x10^3$	$1.53x10^3$
	4	$2.00x10^3$	$2.00x10^3$	$1.00x10^3$	$3.00x10^3$	$1.00x10^3$
	5	$1.67x10^3$	$2.00x10^3$	$1.00x10^3$	$2.00x10^3$	$5.77x10^2$
	6	$1.67x10^3$	$2.00x10^3$	$1.00x10^3$	$2.00x10^3$	$5.77x10^2$
	7	$1.67x10^3$	$2.00x10^3$	$1.00x10^3$	$2.00x10^3$	$5.77x10^2$
	8	$1.83x10^3$	$1.90x10^3$	$1.20x10^3$	$2.40x10^3$	$6.03x10^2$
Haemolytic Staphylococcus	1	$2.87x10^4$	$2.80x10^4$	$9.00x10^3$	$5.20x10^4$	$1.51x10^4$
	2	$9.73x10^4$	$9.90x10^4$	$9.40x10^4$	$9.90x10^4$	$2.89x10^3$
	3	$5.13x10^4$	$5.10x10^4$	$4.80x10^4$	$5.50x10^4$	$3.51x10^3$
	4	$3.77x10^4$	$3.80x10^4$	$3.50x10^4$	$4.00x10^4$	$2.52x10^3$
	5	$5.80x10^4$	$5.50x10^4$	$4.10x10^4$	$7.80x10^4$	$1.87x10^4$
	6	$1.93x10^4$	$1.50x10^4$	$1.40x10^4$	$2.90x10^4$	$8.39x10^3$
	7	$5.67x10^4$	$5.10x10^4$	$5.00x10^4$	$6.90x10^4$	$1.07x10^4$
	8	$5.40x10^4$	$5.50x10^4$	$4.90x10^4$	$5.80x10^4$	$4.58x10^3$
Enterobacteriaceae	1	$6.80x10^3$	$6.15x10^3$	$3.20x10^3$	$1.05x10^4$	$2.89x10^3$
	2	$6.17x10^3$	$6.10x10^3$	$5.80x10^3$	$6.60x10^3$	$4.04x10^2$
	3	$1.28x10^4$	$1.29x10^4$	$9.40x10^3$	$1.60x10^4$	$3.30x10^3$
	4	$2.33x10^3$	$2.20x10^3$	$2.10x10^3$	$2.70x10^3$	$3.21x10^2$
	5	$2.74x10^4$	$2.36x10^4$	$2.26x10^4$	$3.60x10^4$	$7.46x10^3$
	6	$8.00x10^2$	$1.10x10^3$	$1.00x10^2$	$1.20x10^3$	$6.08x10^2$
	7	$2.67x10^2$	$3.00x10^2$	$2.00x10^2$	$3.00x10^2$	$5.77x10^1$
	8	$3.33x10^2$	$3.00x10^2$	$3.00x10^2$	$4.00x10^2$	$5.77x10^1$
Pseudomonas fluorescens	1	$4.17x10^1$	$3.00x10^1$	$1.00x10^1$	$1.00x10^2$	$3.82x10^1$
	2	$2.33x10^1$	$2.00x10^1$	$2.00x10^1$	$3.00x10^1$	$5.77x10^0$
	3	$1.73x10^3$	$1.70x10^3$	$1.60x10^3$	$1.90x10^3$	$1.53x10^2$
	4	$6.33x10^1$	$6.00x10^1$	$6.00x10^1$	$7.00x10^1$	$5.77x10^0$
	5	$9.90x10^2$	$2.40x10^2$	$2.30x10^2$	$2.50x10^3$	$1.31x10^3$
	6	$2.37x10^3$	$2.30x10^3$	$2.20x10^3$	$2.60x10^3$	$2.08x10^2$
	7	$2.67x10^2$	$2.00x10^2$	$2.00x10^2$	$4.00x10^2$	$1.15x10^2$
	8	$1.67x10^2$	$1.00x10^2$	$1.00x10^2$	$3.00x10^2$	$1.15x10^2$
Sulfate reducing anaerobes	1	$2.78x10^1$	$3.00x10^1$	$1.80x10^1$	$3.30x10^1$	$5.85x10^0$
	2	$1.33x10^2$	$1.30x10^2$	$1.28x10^2$	$1.40x10^2$	$6.43x10^0$
	3	$5.43x10^1$	$5.10x10^1$	$4.40x10^1$	$6.80x10^1$	$1.23x10^1$
	4	$5.77x10^1$	$5.50x10^1$	$3.70x10^1$	$8.10x10^1$	$2.21x10^1$
	5	$1.12x10^2$	$1.02x10^2$	$8.80x10^1$	$1.45x10^2$	$2.97x10^1$
	6	$4.30x10^1$	$4.10x10^1$	$3.30x10^1$	$5.50x10^1$	$1.11x10^1$

7	$8.63x10^1$	$8.10x10^1$	$8.00x10^1$	$9.80x10^1$	$1.01x10^1$
8	$7.70x10^1$	$7.70x10^1$	$7.00x10^1$	$8.40x10^1$	$7.00x10^0$
