# Supplementary material for: Uncontrolled Post-Industrial Landfill—Source of Metals, Potential Toxic Compounds, Dust, and Pathogens in Environment—A Case Study
Source: Molecules. 2024 Mar 27;29(7):1496. doi: 10.3390/molecules29071496 (PMC11013361; doi:10.3390/molecules29071496)
Supplement: Supplementary file 1 [file molecules-29-01496-s001.zip › Table S6.pdf]

Table S6. IC_{50} [mg/mL] values of soil extracts against cell lines.

Sample	Cell Line			
	**A-549**	**Hep-G2**	**Caco-2**	**IEC-6**
S1	nd	nd	nd	72.66
S2	nd	nd	nd	92.19
S3	nd	nd	nd	79.77
S4	nd	nd	nd	82.25
S5	nd	nd	nd	92.54
S6	nd	nd	85.81	29.24
S7	nd	nd	nd	nd
S8	nd	nd	nd	nd

nd- not detected
