# Supplementary figures and images for: Uncontrolled Post-Industrial Landfill—Source of Metals, Potential Toxic Compounds, Dust, and Pathogens in Environment—A Case Study
Source: Molecules. 2024 Mar 27;29(7):1496. doi: 10.3390/molecules29071496 (PMC11013361; doi:10.3390/molecules29071496)

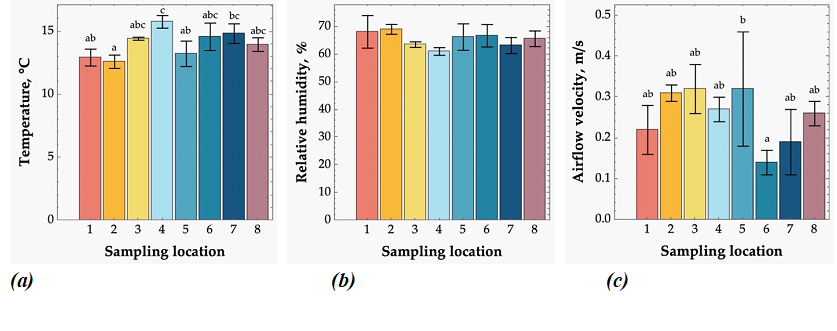

Supplement: Supplementary file 1 [file molecules-29-01496-s001.zip › Figure S1.tif]
